# Molecular insights into electroreceptor ribbon synapses from differential gene expression in sturgeon lateral line organs

**DOI:** 10.1111/joa.70061

**Published:** 2025-11-21

**Authors:** Alexander S. Campbell, Martin Minařík, David Buckley, Tanmay Anand, David Gela, Martin Pšenička, Clare V. H. Baker

**Affiliations:** ^1^ Department of Physiology, Development & Neuroscience University of Cambridge Cambridge UK; ^2^ Departamento de Biología (C. D. Genética), Facultad de Ciencias Universidad Autónoma de Madrid (UAM) Madrid Spain; ^3^ Faculty of Fisheries and Protection of Waters, Research Institute of Fish Culture and Hydrobiology University of South Bohemia in České Budějovice Vodňany Czech Republic; ^4^ Present address: Wellcome Sanger Institute Cambridge UK

**Keywords:** ampullary organ, *Apba1*, *Cbln18*, *Dscaml1*, EAAT1, electroreceptor, hair cell, lateral line, neuromast, *Nrxn3*, *Otof*, *Pcp4*, *Rab3a*, ribbon synapse, *Slc17a8*, *Slc1a3*, sterlet sturgeon, *Syt14*, *Tulp1*, vGlut3

## Abstract

In fishes and aquatic‐stage amphibians, mechanosensory neuromasts are arranged in characteristic lines in the skin of the head and trunk, with afferent innervation from anterior or posterior lateral line nerves. In electroreceptive non‐teleost jawed fishes and amphibians, fields of electrosensory ampullary organs flank some or all of the cranial neuromast lines, innervated by the anterior lateral line nerve. Like the mechanosensory hair cells found in neuromasts and the inner ear, electroreceptor cells in ampullary organs across vertebrates form specialised ribbon synapses with afferent nerve terminals. Ribbon synapses in hair cells are distinct from other glutamatergic synapses, including the ribbon synapses in photoreceptors: In hair cells, synaptic vesicles are loaded with glutamate by vGlut3 and otoferlin is the Ca^2+^ sensor for synaptic vesicle exocytosis. We previously showed that the genes encoding vGlut3 and otoferlin are expressed by ampullary organs as well as neuromasts in a chondrostean ray‐finned fish, the Mississippi paddlefish (*Polyodon spathula*), suggesting that electroreceptor ribbon synapses are very similar to those in hair cells. In this study, we selected additional synapse‐related candidate genes from our previously published dataset of putatively lateral line organ‐enriched genes from late‐larval paddlefish, and examined their expression in developing lateral line organs in a more experimentally tractable chondrostean, the sterlet sturgeon (*Acipenser ruthenus*). We found that sterlet ampullary organs express genes encoding vGlut3 (as expected from paddlefish) and the high‐affinity glutamate re‐uptake transporter EAAT1 (GLAST). Sterlet ampullary organs also express *Otof* (also expected from paddlefish, though we identified one *Otof* transcript variant maintained in ampullary organs but not neuromasts) and two other hair cell synapse‐associated genes, *Apba1* (*Mint1*) and *Rab3a*. Genes encoding the presynaptic cell adhesion molecule Nrxn3, the calcium‐independent synaptotagmin Syt14, the calmodulin regulator protein PCP4 (PEP‐19) and cell adhesion molecule DSCAML1 were expressed in both neuromasts and ampullary organs. In contrast, *Cbln18*, encoding a secreted trans‐synaptic scaffolding protein, was only expressed in neuromasts and *Tulp1*, encoding tubby‐related protein 1 (required for the development and function of photoreceptor ribbon synapses), was only expressed in ampullary organs. Overall, our results support electroreceptor ribbon synapses in non‐teleost ray‐finned bony fish being glutamatergic and suggest further commonalities, but also some differences, with hair cell ribbon synapses.

## Graphical abstract

The expression of various genes involved in synapse development, stability and/or function was studied in developing mechanosensory neuromasts and electrosensory ampullary organs in sterlet sturgeon. Most were shared, including *Slc17a8*, *Slc1a3* and *Nrxn3*, but *Cbln18* was neuromast‐restricted and *Tulp1* was ampullary organ‐restricted. The results support electroreceptor ribbon synapses being glutamatergic and suggest further similarities, but also some differences, with hair cell ribbon synapses.

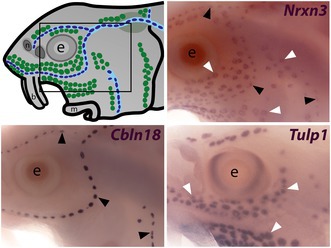

## 1 INTRODUCTION

Fishes and aquatic‐stage amphibians possess an evolutionarily ancient sensory system known as the lateral line (Bullock et al., 1983; Mogdans, 2020; Northcutt, 1997). Neuromasts in the skin, distributed in lines over the head and trunk, contain mechanosensory hair cells that respond to nearby water movement (‘touch at a distance’), used for detecting prey or predators, obstacles and orientation (Mogdans, 2020; Montgomery et al., 2014). Mechanical deflection of the apical ‘hair bundle’ of stepped microvilli (stereocilia) leads to cation entry via the mechanoelectrical transduction complex, depolarising the cell (Deng & Yan, 2025; Holt et al., 2024). The hair cells in neuromasts are very similar to vestibular inner‐ear hair cells (Nicolson, 2017; Shi et al., 2023). In electroreceptive non‐teleost jawed fishes and amphibians, at least some neuromast lines are flanked by electrosensory ampullary organs containing electroreceptor cells (Baker et al., 2013; Bullock et al., 1983; Crampton, 2019; Elliott & Fritzsch, 2020). These have voltage‐gated calcium channels in the apical membrane (identified as Ca_v_1.3 in sharks and skates; Bellono et al., 2017; Bellono et al., 2018) that respond to low‐frequency cathodal (exterior negative) environmental stimuli, such as electric fields around other animals in water, primarily for detecting prey or predators (Bodznick & Montgomery, 2005; Bullock et al., 1983; Crampton, 2019). Ampullary organs across jawed vertebrates are homologous (i.e. share a common ancestor), as shown by conserved embryological origin from lateral line placodes and shared gene expression in representatives of cartilaginous fishes, ray‐finned bony fishes and lobe‐finned bony tetrapods (Baker et al., 2013; Baker & Modrell, 2018; Baker, 2019).

Afferent innervation for hair cells is provided by neurons in cranial lateral line ganglia, projecting via the anterior or posterior lateral line nerves (depending on the position of the neuromast) to the medial octavolateral nucleus of the hindbrain (Elliott & Fritzsch, 2020; Wullimann & Grothe, 2014). Electroreceptor afferents are provided exclusively by the anterior lateral line nerve, projecting centrally via its dorsal root to the dorsal octavolateral nucleus of the hindbrain (Bullock et al., 1983; Elliott & Fritzsch, 2020; Wullimann & Grothe, 2014). Across vertebrates, both hair cells and electroreceptor cells (Elliott & Fritzsch, 2020; Jørgensen, 2005) form specialised ribbon synapses with afferent nerve terminals, characterised by an electron‐dense presynaptic ‘ribbon’ tethering a readily releasable pool of synaptic vesicles, enabling response to graded signals and sustained neurotransmitter release (Johnson et al., 2019; Moser et al., 2020; Nicolson, 2015). Presynaptic ribbons in hair cells may be round, ovoid, wedge‐shaped or plate‐like, and ribbons of different sizes can be found within the same hair cell (Moser et al., 2020). Those in electroreceptors vary greatly in shape and size across different species, from sheets (for example, in paddlefish, sturgeon, bichir and skate species; Jørgensen et al., 1972; Jørgensen, 1980; Jørgensen, 1982; Sejnowski & Yodlowski, 1982) to rounded bodies of varying diameter, from 0.2 μm in lampreys (Whitear & Lane, 1981) to 3 μm in a newt species (mean diameter 2 μm in electroreceptors versus 0.8 μm in hair cells; Fritzsch & Wahnschaffe, 1983).

In both hair cells and electroreceptors, depolarisation in response to stimulation opens voltage‐gated calcium channels (Ca_v_1.3 in hair cells) clustered at ribbon synapses, leading to synaptic vesicle exocytosis and release of neurotransmitter (glutamate in hair cells) (Bodznick & Montgomery, 2005; Johnson et al., 2019; Moser et al., 2020; Nicolson, 2015). Ca_v_1.3 has also been identified as the voltage‐sensing channel in the apical membrane of shark and skate electroreceptors (Bellono et al., 2017; Bellono et al., 2018; reviewed by Leitch & Julius, 2019). In hair cells, calcium entry via ribbon synapse‐associated Ca_v_1.3 channels triggers synaptic vesicle exocytosis by binding to otoferlin, a transmembrane Ca^2+^ sensor that interacts directly with Ca_v_1.3 and membrane‐fusion machinery specifically in hair cells (Johnson et al., 2019; Leclère & Dulon, 2023; Moser et al., 2020). Hair cell ribbon synapses are also unique in that their synaptic vesicles are filled with glutamate by the vesicular glutamate transporter vGlut3, whereas photoreceptor ribbon synapses use vGlut1 and vGlut2 (Johnson et al., 2019; Moser et al., 2020). However, very little information is available about the molecular nature of electroreceptor ribbon synapses.

We previously reported the expression of *Cacna1d* (encoding the pore‐forming subunit of Ca_v_1.3), *Otof* (encoding otoferlin) and *Slc17a8* (encoding vGlut3) in late‐larval ampullary organs as well as neuromasts in a chondrostean ray‐finned fish, the Mississippi paddlefish (*Polyodon spathula*) (Modrell, Lyne, et al., 2017). These genes were identified via a differential RNA‐seq screen in late‐larval paddlefish comparing the transcriptomes of operculum (rich in ampullary organs, plus some neuromasts) versus fin (similar tissue composition, but without lateral line organs) (Modrell, Lyne, et al., 2017). VGlut3 protein expression in juvenile paddlefish electroreceptors was also recently reported by immunolabelling (Russell et al., 2025). The conserved expression of these genes in paddlefish ampullary organs and neuromasts suggested that electroreceptor ribbon synapses operate in the same way as hair cell ribbon synapses. We also identified a few genes likely to be important specifically for electroreceptor function: Two voltage‐gated potassium channel genes (*Kcna5* and *Kcnab3*) and a beta‐parvalbumin gene (encoding a calcium‐buffering protein) were expressed in paddlefish ampullary organs but not in neuromasts (Modrell, Lyne, et al., 2017). We also found conserved expression of many developmental genes, suggesting that electroreceptors are closely related to hair cells, as well as some developmental genes (encoding transcription factors and signalling pathway components) expressed specifically in either ampullary organs or neuromasts (Campbell et al., 2025; Minařík et al., 2024; Minařík et al., 2025; Modrell, Lyne, et al., 2017; Modrell, Tidswell, & Baker, 2017).

Here, we aimed to shed further light on the molecular nature of ribbon synapses in electroreceptors and their conservation with hair cell ribbon synapses. To do so, we examined the expression in developing neuromasts and/or ampullary organs of additional synapse‐related candidate genes from our late‐larval paddlefish dataset of ~500 putatively lateral line organ‐enriched genes (Modrell, Lyne, et al., 2017), in a more experimentally tractable chondrostean model, the sterlet sturgeon (*Acipenser ruthenus*; see Minařík et al., 2024; Minařík et al., 2025; Campbell et al., 2025). We previously confirmed in sterlet that, as expected from paddlefish (Modrell, Lyne, et al., 2017), both neuromasts and ampullary organs express *Cacna1d* but only ampullary organs express *Kcnab3* (Minařík et al., 2024). The new gene expression data reported here support extensive molecular conservation between hair cell and electroreceptor ribbon synapses in non‐teleost ray‐finned bony fishes, including the use of glutamate as the neurotransmitter, but also identify some molecular differences.

## 2 METHODS

### 2.1 Collection, staging and fixation of sterlet yolksac larvae

Yolksac larvae of sterlet sturgeon (*Acipenser ruthenus*) were obtained at the Research Institute of Fish Culture and Hydrobiology, Faculty of Fisheries and Protection of Waters, University of South Bohemia in České Budějovice (Vodňany, Czech Republic). Stundl et al. (2022) provide detailed information about adult sterlet husbandry, in vitro fertilisation and raising embryos and yolksac larvae. Larvae were staged according to Dettlaff et al. (1993). The earliest stage used was Stage 36 (hatching; 6 days post fertilisation [dpf]; Zeiske et al., 2003), which is shortly after the first *Cacna1d*‐positive differentiated hair cells are seen in cranial neuromasts at Stage 35 (Minařík et al., 2024). The first differentiated electroreceptors (identified by *Cacna1d* or *Kcnab3* expression in ampullary organs) are seen a few days later, at Stages 40–41 (Minařík et al., 2024). The latest stage used was Stage 45 (the onset of independent feeding at 14 dpf; Zeiske et al., 2003), when all ampullary organ fields are developed (Minařík et al., 2024). Following euthanasia by anaesthetic overdose with MS‐222 (Sigma‐Aldrich), larvae were fixed for 3 h at room temperature in modified Carnoy's fixative (6 volumes 100% ethanol: 3 volumes 37% formaldehyde: 1 volume glacial acetic acid) and graded into ethanol before storing at −20°C.

All experimental procedures were approved by the Animal Research Committee of the Faculty of Fisheries and Protection of Waters in Vodňany, University of South Bohemia in České Budějovice, Czech Republic, and by the Ministry of Agriculture of the Czech Republic (reference number: MSMT‐12550/2016‐3). Experimental fish were maintained according to the principles of the European Union (EU) Harmonized Animal Welfare Act of the Czech Republic, and Principles of Laboratory Animal Care and National Laws 246/1992 ‘Animal Welfare’ on the protection of animals.

### 2.2 Gene cloning and riboprobe templates

To make late‐larval sterlet cDNA, Trizol (Invitrogen, Thermo Fisher Scientific) was used to extract total RNA from Stage 45 sterlet larval heads. After treating with DNAse using the Ambion Turbo DNA‐free kit (Invitrogen, Thermo Fisher Scientific), cDNA was synthesised using the High‐Capacity cDNA Reverse Transcription Kit (Applied Biosystems), following the manufacturers' instructions. Genes were selected from the late‐larval paddlefish (*Polyodon spathula*) lateral line organ‐enriched gene set (National Center for Biotechnology Information [NCBI] Gene Expression Omnibus (RRID:SCR_005012) accession code GSE92470; Modrell, Lyne, et al., 2017). Using a Basic Local Alignment Search Tool (BLAST) database generated from our sterlet transcriptome assemblies (from pooled Stages 40–45 larval sterlet heads; Minařík et al., 2024), which are available at DDBJ/EMBL/GenBank under the accessions GKLU00000000 and GKEF01000000, the appropriate paddlefish transcriptome sequence was used in a command‐line search to identify homologous sterlet sequences. NCBI BLAST (https://blast.ncbi.nlm.nih.gov/Blast.cgi; RRID:SCR_004870; McGinnis & Madden, 2004) was used to check sterlet sequence identity. PCR primers (Table S1) were designed with Primer3Plus (RRID:SCR_003081; Untergasser et al., 2012), which is also incorporated into the Editor program of Benchling (https://benchling.com; RRID:SCR_013955). The primers were used to amplify cDNA fragments from sterlet cDNA under standard PCR conditions. After cloning the cDNA fragments into Qiagen's pDrive cloning vector using the Qiagen PCR Cloning Kit (Qiagen), the clones were checked by sequencing (Department of Biochemistry Sequencing Facility, University of Cambridge). Sequence identity was confirmed using NCBI BLAST. For *Apba1*, *Dscam*, *Dscaml1*, *Otof*, *Rab3a* and *Syt14*, synthetic gene fragments with added M13 forward and reverse primer adaptors were designed using sterlet transcriptome data and bought from Twist Bioscience.

Most of these sterlet riboprobe template sequences were designed before the publication of chromosome‐level genome assemblies for sterlet: Du et al. (2020) and the 2022 NCBI RefSeqGene (RRID:SCR_013787) reference genome assembly (GCF_902713425.1/). For all genes described here except *Cbln18*, both ohnologs (i.e. gene paralogs resulting from the whole‐genome duplication) have been retained from the whole‐genome duplication that occurred in the chondrostean lineage (Du et al., 2020; Redmond et al., 2023). Our phylogenetic analysis of *Cbln* family genes showed that the single *Cbln18* ohnolog in the reference sterlet genome (GCF_902713425.1) has been mis‐annotated as *Cbln3* (Figure 2; Table S1). Table S1 includes the percentage match of each riboprobe with each ohnolog, obtained by using NCBI BLAST to perform a nucleotide BLAST search against the reference genome assembly (NCBI RefSeqGene assembly GCF_902713425.1/). The percentage match with the ‘targeted’ ohnolog ranged from 97.8 to 100% (Table S1). Apart from *Dscaml1* and *Otof*, the percentage match with the second ohnolog (where present) ranged from 94.4 to 100% (Table S1), suggesting that transcripts from the second ohnolog are also targeted by our riboprobes. Table S1 also gives the GenBank (RRID:SCR_002760) accession numbers for each riboprobe's top match, and the nucleotide range targeted by each riboprobe.

### 2.3 Phylogenetic analysis of cerebellins

Orthologs of the target sequence (encoded by the gene annotated *Cbln3* in the sterlet reference genome assembly; GCF_902713425.1/) were checked in the SHOOT.bio database (https://shoot.bio; Emms & Kelly, 2022). The target sequence clustered with different cerebellin 18 sequences. To confirm its nature, amino acid sequences from different cerebellin families (104 vertebrate sequences plus the sole amphioxus cerebellin sequence; Table S2) were downloaded from GenBank (RRID:SCR_002760) and aligned using the online version of MAFFT 7 (https://mafft.cbrc.jp/alignment/server/; RRID:SCR_011811; Katoh et al., 2019) with the G‐INS‐i iterative refinement method. Two Bayesian phylogenetic analyses were run with the aligned data matrix. First, a search in MrBayes v.3.2.7 (RRID:SCR_012067; Ronquist et al., 2012) was performed: two analyses of 10 million MC^3^ chains were run, sampling the chains every 1000 generations. These were averaged over all possible substitution models with the option ‘aamodelpr=mixed’. Trees were rooted with mid‐point rooting. The second Bayesian phylogenetic analysis was run in PhyloBayes MPI v1.8c (RRID:SCR_006402; Lartillot et al., 2013) in the server CIPRES Science Gateway (https://www.phylo.org/; RRID:SCR_008439; Miller et al., 2010) under the infinite mixture CAT‐GTR model. The tree was rooted with mid‐point rooting. The resulting trees were visualised and edited in FigTree v1.4.4 (RRID:SCR_008515; Rambaut, 2018) and Adobe Photoshop (RRID:SCR_014199; Adobe Systems Inc.).

### 2.4 In situ hybridisation

Digoxigenin‐labelled riboprobes were prepared as previously described (Minařík et al., 2024). Larvae were bleached and wholemount in situ hybridisation (ISH) was performed as described in Modrell et al. (2011). At least three larvae were used per stage.

### 2.5 Image capture and processing

Sterlet larvae were imaged using either a QImaging MicroPublisher 5.0 RTV camera controlled by the QCapture Pro 7.0 software (RRID:SCR_014432; QImaging) or a MicroPublisher 6 colour CCD camera (Teledyne Photometrics) controlled by Ocular software (RRID:SCR_024490; Teledyne Photometrics), fitted to a Leica MZFLIII dissecting microscope. Helicon Focus software (RRID:SCR_014462; Helicon Soft Limited) was used for focus stacking of image stacks collected by focusing manually through the sample. Images were processed with Adobe Photoshop (RRID:SCR_014199; Adobe Systems Inc.).

## 3 RESULTS

We aimed to gain further insight into the likely molecular nature of electroreceptor ribbon synapses by studying the expression in developing ampullary organs and neuromasts of additional synapse‐related genes identified by manual review of our late‐larval paddlefish lateral line organ‐enriched gene set (Modrell, Lyne, et al., 2017). For this study, as in our other recent work on ampullary organ development using genes from the paddlefish gene set (Campbell et al., 2025; Minařík et al., 2024; Minařík et al., 2025), we used a more experimentally tractable chondrostean model, namely, the sterlet sturgeon (*A. ruthenus*). The latest stage examined was Stage 45, the onset of independent feeding (14 days post fertilisation; Zeiske et al., 2003), when all ampullary organ fields are developed (Minařík et al., 2024). At the onset of active feeding in a related sturgeon, *A. naccarii*, the ultrastructural characteristics of ampullary organs, including ribbon synapses, already correspond to those of adult ampullary organs (Camacho et al., 2007).

### 3.1 *Slc17a8*, encoding vGlut3, and *Slc1a3*, encoding excitatory amino acid transporter 1 (EAAT1), are expressed in sterlet neuromasts and ampullary organs

**FIGURE 1:**
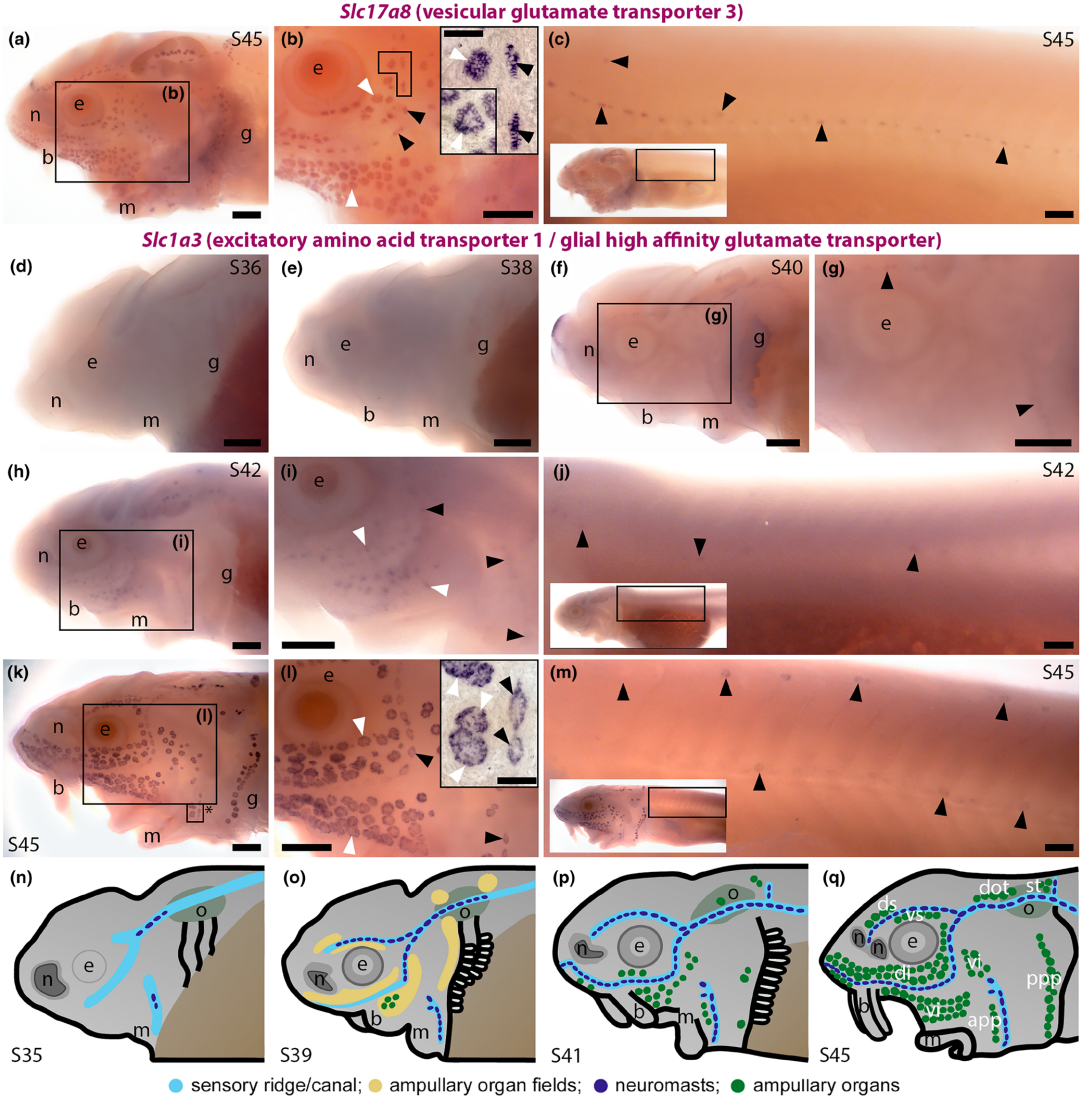
Sterlet *Slc17a8* (encoding vGlut3) and *Slc1a3* (encoding EAAT1/GLAST) are expressed in neuromasts and ampullary organs. In situ hybridisation in sterlet. Black arrowheads indicate examples of neuromasts; white arrowheads indicate examples of ampullary organs. For images of the trunk, boxes on low‐power insets delineate the location of the trunk regions shown. (a–c) At Stage 45 (the onset of independent feeding), *Slc17a8* expression is seen in ampullary organs and neuromasts on the head (a, b) and neuromasts on the trunk (c), as previously reported for paddlefish. The inset in panel b shows a skin mount from the region delineated by the box and another from a different area (on the other side of the head). (d, e) *Slc1a3* expression is not seen at Stage 36 (d) or Stage 38 (e). (f, g) At Stage 40, *Slc1a3* expression is observed in cranial neuromasts. (h–j) At Stage 42, *Slc1a3* expression persists in cranial neuromasts and is also seen in ampullary organs (h, i) and trunk neuromasts (j). (k–m) At Stage 45, *Slc1a3* expression persists on the head in ampullary organs and neuromasts (k, l) and more weakly in trunk neuromasts. The ring‐like expression pattern in neuromasts (seen most clearly in cranial neuromasts, panel l; inset shows a skin mount from the boxed region indicated by the asterisk in panel k) suggests *Slc1a3* is expressed by supporting cells rather than hair cells (compare with *Slc17a8* expression in neuromasts in the skin mount in panel b). (n–q) Schematic illustrations of sterlet lateral line organ development at similar stages (previously published in Minařík et al., 2024). Abbreviations: app, anterior preopercular ampullary organ field; b, barbel; di, dorsal infraorbital ampullary organ field; dot, dorsal otic ampullary organ field; ds, dorsal supraorbital ampullary organ field; e, eye; g, gill filaments; m, mouth; n, naris; o, otic vesicle; ppp, posterior preopercular ampullary organ field; S, stage; st, supratemporal ampullary organ field; vi, ventral infraorbital ampullary organ field; vs, ventral supraorbital ampullary organ field. Scale bar: 250 μm.

We previously showed by wholemount in situ hybridisation (ISH) in paddlefish that *solute carrier family 17 member 8* (*Slc17a8*), encoding vGlut3 (which was 30.9‐fold enriched in late‐larval paddlefish operculum versus fin tissue; Modrell, Lyne, et al., 2017), is expressed by ampullary organs as well as neuromasts at Stage 46, the onset of independent feeding (Modrell, Lyne, et al., 2017). As expected, given that both paddlefish and sterlet are chondrostean fishes, *Slc17a8* was also expressed by sterlet ampullary organs at the onset of independent feeding (Stage 45; Figure 1a–c).

*Solute carrier family 1 member 3* (*Slc1a3*), encoding excitatory amino acid transporter 1 (EAAT1), also known as glial high‐affinity glutamate transporter and glutamate/aspartate transporter (GLAST), was 11.3‐fold lateral line organ‐enriched in late‐larval paddlefish (Modrell, Lyne, et al., 2017). In the CNS, EAAT1 is found in the membrane of astrocytes where it acts to clear glutamate from the synaptic cleft after synaptic transmission (reviewed by Niciu et al., 2012; Andersen et al., 2021). In the rodent inner ear, EAAT1 is expressed at the basolateral membranes of cochlear and vestibular supporting cells (Furness & Lehre, 1997; Takumi et al., 1997) and is required for glutamate uptake from cochlear hair cell ribbon synapses (Chen et al., 2010; Glowatzki et al., 2006). Zebrafish *slc1a3* is also expressed in neuromasts (Gesemann et al., 2010).

Preliminary in situ hybridisation (ISH) data from late‐larval paddlefish (not shown) identified the expression of *Slc1a3* in both neuromasts and ampullary organs. We undertook a more detailed study in sterlet. *Slc1a3* was not detectably expressed by ISH at Stage 36 (when some differentiated *Cacna1d*‐positive neuromast hair cells are already present; Minařík et al., 2024) or Stage 38 (Figure 1d,e). However, by Stage 40, faint expression was seen in cranial neuromasts only (Figure 1f,g). At this stage, some differentiated electroreceptors are already detectable via *Cacna1d* or *Kcnab3* in ampullary organs (Minařík et al., 2024). At Stage 42, *Slc1a3* expression had extended to ampullary organs (Figure 1h,i) and trunk neuromasts (Figure 1j). At Stage 45, strong expression was seen in ampullary organs and weaker expression in neuromasts on both head and trunk (Figure 1k–m). Neuromast expression was in a ring‐like pattern (compare skin‐mount *Slc1a3* expression in Figure 1l with skin‐mount *Slc17a8* expression in Figure 1b), suggesting *Slc1a3* is expressed by supporting cells. These data suggest that EAAT1 (GLAST) expressed by supporting cells may clear glutamate from the ribbon synapses of both hair cells and electroreceptors.

Figure 1n–q show schematics (previously published in Minařík et al., 2024) illustrating the progression of cranial neuromast and ampullary organ development in sterlet.

### 3.2 Otoferlin is expressed in sterlet neuromasts and ampullary organs, though one transcript variant is maintained only in ampullary organs

**FIGURE 2:**
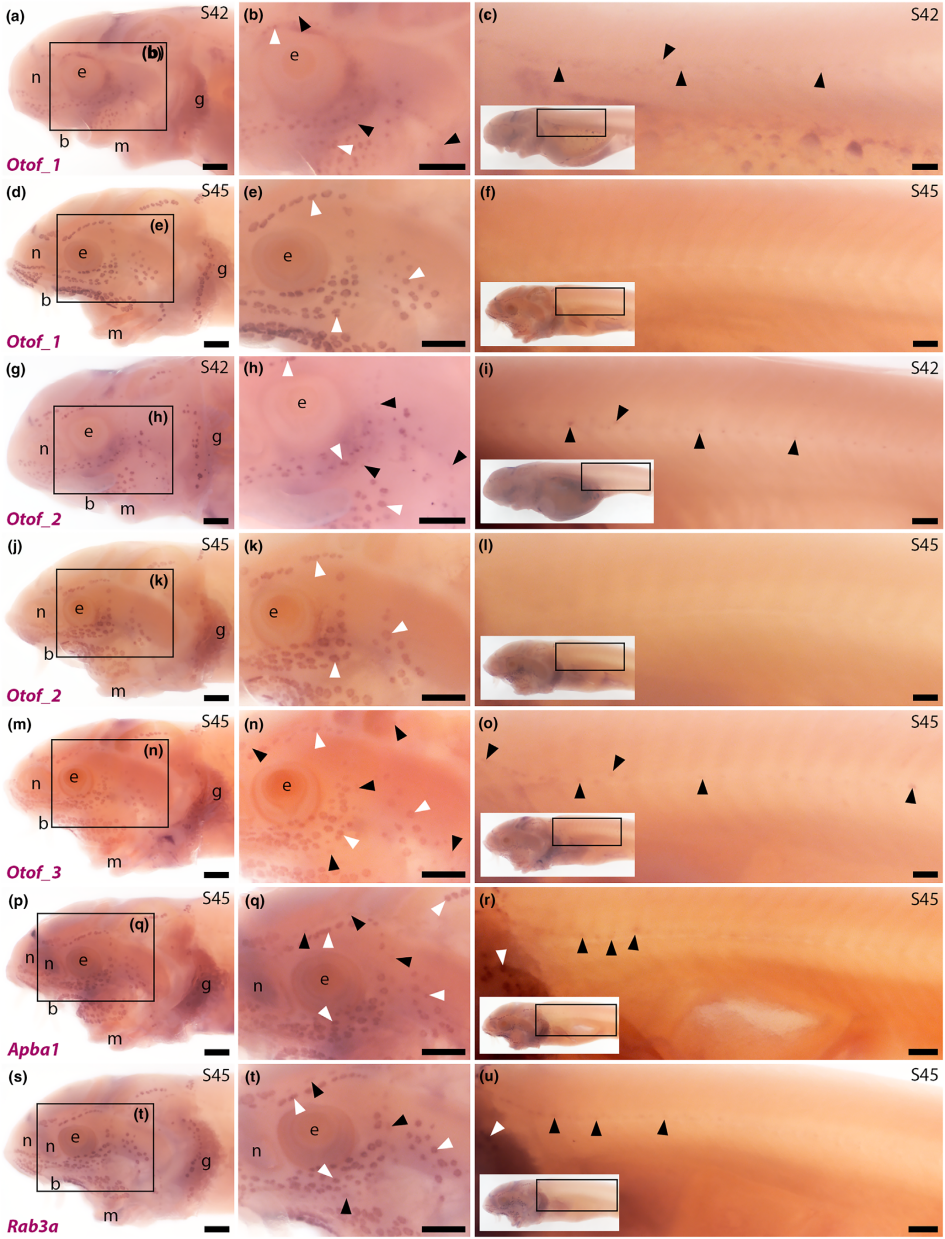
Sterlet ampullary organs express genes encoding the hair cell ribbon synapse‐associated proteins otoferlin, Rab3a and Apba1. In situ hybridisation in sterlet. Black arrowheads indicate examples of neuromasts; white arrowheads indicate examples of ampullary organs. For images of the trunk, boxes on low‐power insets delineate the location of the trunk regions shown. (a–f) The *Otof_1* riboprobe, which targets a transcript region spanning Exons 1–9 of the chromosome‐5 *Otof* ohnolog (Figure 3) reveals expression in ampullary organs and, more weakly, in neuromasts at Stage 42 (a–c), but only in ampullary organs at Stage 45 (d–f). (g–l) The *Otof_2* riboprobe, which specifically targets the ‘longer’ chromosome‐5 *Otof* splice variant that includes Exons 6–10 (Figure 3) also reveals expression in ampullary organs and, more weakly, in neuromasts at Stage 42 (g–i) but only in ampullary organs at Stage 45 (j–l). (m–o) The *Otof_3* riboprobe, which specifically targets the ‘shorter’ *Otof* splice variant produced by both ohnologs, which lacks the equivalent sequence to chromosome‐5 Exons 6–10 (see Figure 3), shows expression in ampullary organs and, more weakly, in neuromasts at Stage 45. (p–r) At Stage 45, *Apba1* is expressed in ampullary organs and, more weakly, in neuromasts. (s–u) At Stage 45, *Rab3a* expression is seen in ampullary organs and, more weakly, in neuromasts. Abbreviations: b, barbel; e, eye; g, gill filaments; m, mouth; n, naris; S, stage. Scale bar: 250 μm.

We previously showed that *Otof* (20.9‐fold lateral line organ‐enriched in late‐larval paddlefish; Modrell, Lyne, et al., 2017), encoding the transmembrane Ca^2+^ sensor otoferlin, is expressed in paddlefish ampullary organs as well as neuromasts at Stage 46 (Modrell, Lyne, et al., 2017). Otoferlin is required for synaptic vesicle release at hair cell ribbon synapses (Johnson et al., 2019; Leclère & Dulon, 2023). We confirmed that sterlet neuromasts and ampullary organs also express *Otof*, as expected from paddlefish (given that both are chondrostean fishes), using a riboprobe designed against an *Otof* transcript from our de novo‐assembled sterlet transcriptomes (from pooled Stages 40–45 sterlet heads; Minařík et al., 2024). ISH using this riboprobe (here designated *Otof_1*) revealed *Otof* expression at Stage 42 in sterlet ampullary organs and, more weakly, in neuromasts (Figure 2a–c). At Stage 45, robust signal persisted in ampullary organs, but expression was no longer seen in neuromasts (Figure 2d–f). This raised the intriguing possibility that different *Otof* transcripts/isoforms are maintained in electroreceptors versus hair cells. There is precedent for this: single‐cell RNA‐sequencing (scRNA‐seq) data in zebrafish show differential expression of *otofa* and *otofb* (i.e. the two gene paralogs resulting from the whole‐genome duplication in the teleost lineage) in different types of hair cells, with *otofa* essentially specific to inner ear hair cells and *otofb* expressed in both inner ear and neuromast hair cells (Shi et al., 2023; also see scRNA‐seq data on the Daniocell website from Sur et al., 2023).

**FIGURE 3:**
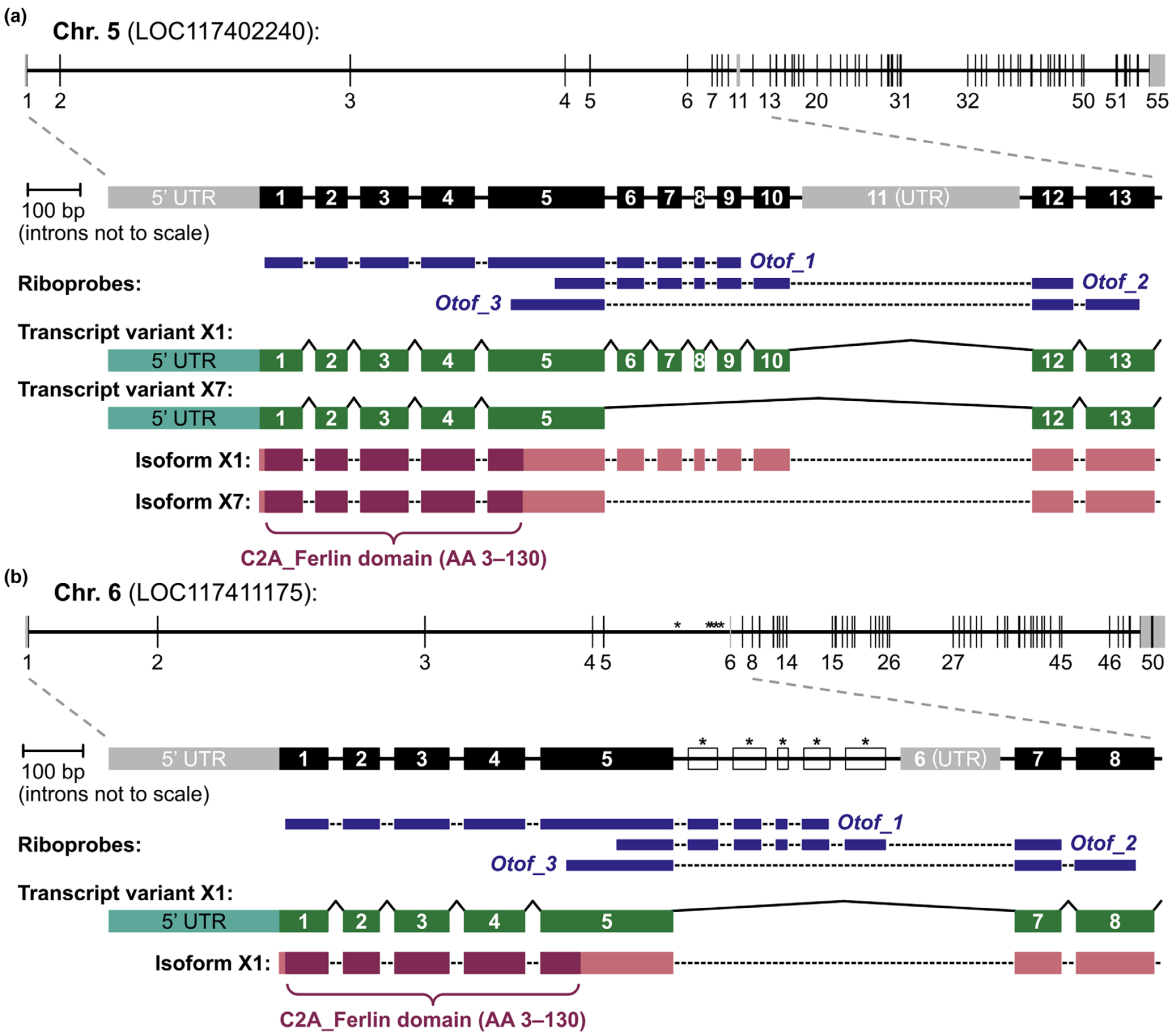
Schematic representation of the two sterlet *Otof* ohnologs, sample transcript variants and riboprobes. (a) Schematic depiction (*top*) of the exon‐intron structure in the sterlet reference genome (NCBI RefSeq GCF_902713425.1) of the sterlet *Otof* gene on chromosome 5 (NCBI Gene LOC117402240). The 55 exons are shown as vertical bars (numbered where possible); grey bars indicate non‐coding exons. Below this is an expanded detail (coding exons in black; non‐coding exons in grey; introns not to scale) of the genomic region spanning exon 1 (which includes 5′ untranslated region, UTR) to exon 13. Following this are schematised depictions of riboprobes *Otof_1*, *Otof_2* and *Otof_3*, then two example transcript variants and the corresponding protein isoforms. The ‘longer’ transcript variant X1 (NCBI RefSeq XM_059023685.1) is targeted by riboprobe *Otof_1* (spanning exons 1–9; 99% match, 691/700, see Table S1) and riboprobe *Otof_2* (spanning part of exon 5 through exon 12; 100% match, Table S1). The ‘shorter’ transcript variant X7 (XM_059023691.1) lacks exons 6–11 and is likely to be targeted by riboprobe *Otof_3* (spanning part of exon 5 plus exon 12 and most of exon 13; 91.5% match, Table S1; this riboprobe was designed against the chromosome‐6 *Otof* sequence; see panel b). The corresponding protein isoforms X1 (NCBI RefSeq XP_058879668.1) and X7 (XP_058879674.1) are shown below the transcript variants with the location of the first C2A_Ferlin domain indicated (amino acids 3–130; encoded by exons 1–4 and part of exon 5). (b) Schematic depiction (*top*) of the exon‐intron structure in the sterlet reference genome (NCBI RefSeq GCF_902713425.1) of the sterlet *Otof* gene on chromosome 6 (NCBI Gene LOC117411175). The 50 exons are shown as vertical bars (numbered where possible); grey bars indicate non‐coding exons. Asterisks indicate the sequence corresponding to chromosome‐5 *Otof* exons 6–10, which is annotated as intronic (compare with panel a). Below this is an expanded detail (coding exons in black; non‐coding exons in grey; introns not to scale) of the equivalent genomic region shown in panel a, with the intronic‐annotated sequence corresponding to chromosome‐5 exons 6–10 shown as unfilled boxes with asterisks (compare with panel a). Following this are schematised depictions of riboprobes *Otof_1*, *Otof_2* and *Otof_3*, then transcript variant X1 (NCBI RefSeq XM_ XM_034018415.3). This ‘shorter’ transcript is unlikely to be targeted by riboprobe *Otof_1* (72% match; 504/700) and will not be targeted by riboprobe *Otof_2* (42% match; 169/400). Riboprobe *Otof_3* was designed to target this transcript (100% match, 349/349). The corresponding protein isoform X1 (NCBI RefSeq XP_033874306.1) is shown below the transcript with the location of the first C2A_Ferlin domain indicated (amino acids 3–130; encoded by exons 1–4 and part of exon 5).

When checked against the sterlet reference genome, the 700‐base *Otof_1* riboprobe designed against our de novo‐assembled transcriptome sequence was a 99% match (100% excluding a 9‐base insert; Table S1) for a 691‐base region of the predicted X1 transcript from the chromosome‐5 *Otof* ohnolog (i.e. one of the two gene paralogs resulting from the whole‐genome duplication in the chondrostean lineage; Du et al., 2020; Redmond et al., 2023). The region of the transcript targeted by the *Otof_1* riboprobe spans exons 1–9 of the *Otof* gene on chromosome 5 (see schematics in Figure 3a). Exons 1–4 plus approximately the first third of exon 5 together encode the N terminus plus the first C2 domain (C2A_Ferlin domain, amino acids 3–130); the remainder of exon 5 plus exons 6–10 encode part of the first inter‐C2 domain (Figure 3a). However, a predicted alternative splice variant for the chromosome‐5 ohnolog lacks exons 6–10 (Figure 3a) and we found a transcript for this ‘shorter’ splice variant in our de novo‐assembled sterlet transcriptome. Furthermore, the *Otof_1* riboprobe only had 72% identity overall to the predicted chromosome‐6 ohnolog X1 transcript (Table S1): the equivalent sequence to chromosome‐5 *Otof* exons 6–10 (encoding part of the first inter‐C2 domain) is annotated as intronic and the predicted chromosome‐6 *Otof* transcripts exclude this region (Figure 3b). This suggests that the ‘longer’ transcript may be specific to the chromosome‐5 ohnolog, whereas the ‘shorter’ splice variant (lacking the equivalent sequence to chromosome‐5 *Otof* exons 6–10) is predicted to be transcribed from both ohnologs (Figure 3a,b).

To target the ‘longer’ chromosome‐5‐specific splice variant (including chromosome‐5 *Otof* exons 6–10), we designed a second riboprobe, *Otof_2* (Figure 3a). ISH using this riboprobe revealed the same expression pattern as the *Otof_1* riboprobe, namely, expression in ampullary organs and, more weakly, in neuromasts at Stage 42 (Figure 2g–i), but only in ampullary organs at Stage 45 (Figure 2j–l). This suggests that the ‘longer’ chromosome‐5‐specific splice variant is maintained in electroreceptors but lost from hair cells.

To target the ‘shorter’ splice variant (i.e. missing the equivalent sequence to chromosome‐5 *Otof* exons 6–10), we designed a third riboprobe, *Otof_3*, against the chromosome‐6 *Otof* sequence (Figure 3a,b). This riboprobe has a 91.5% match to the equivalent region from the chromosome‐5 ohnolog (Table S1), so it will most likely target transcripts from both ohnologs (Figure 3a,b). ISH using the *Otof_3* riboprobe at Stage 45 revealed weak expression in neuromasts as well as stronger expression in ampullary organs (Figure 2m–o). Hence, the ‘shorter’ splice variant (transcribed from both ohnologs) is maintained in neuromasts as well as ampullary organs.

Overall, we found that, as expected from paddlefish (Modrell, Lyne, et al., 2017), sterlet ampullary organs express *Otof*, suggesting that otoferlin's function in neurotransmitter release at the hair cell ribbon synapse (Johnson et al., 2019; Leclère & Dulon, 2023) is conserved at the electroreceptor ribbon synapse. However, the ampullary organ‐specific expression of one *Otof* splice variant at Stage 45 (the onset of independent feeding) suggests some degree of specialisation, or subfunctionalisation, of otoferlin variants in electroreceptor versus hair cell ribbon synapses.

### 3.3 Sterlet ampullary organs express genes encoding the hair cell ribbon synapse‐associated proteins Apba1 (X11α/Mint1) and Rab3a

We designed riboprobes targeting five other genes associated with hair cell ribbon synapses that were present in the paddlefish lateral line organ‐enriched gene set (Modrell, Lyne, et al., 2017), but ISH using three of these riboprobes was unsuccessful. These were: *Snap25* (6.9‐fold lateral line organ‐enriched; Modrell, Lyne, et al., 2017), encoding synaptosome associated protein 25, which is essential for normal synaptic vesicle exocytosis in cochlear hair cells (Calvet et al., 2022); *Snap91* (3.0‐fold lateral line organ‐enriched; Modrell, Lyne, et al., 2017), encoding synaptosome associated protein 91 (also known as adaptor protein AP180), which is essential for synaptic vesicle recycling in cochlear inner hair cells (Kroll et al., 2020); and *Amph*, encoding the endocytic protein amphiphysin (4.0‐fold lateral line organ‐enriched; Modrell, Lyne, et al., 2017), which is expressed in cochlear inner hair cells (Neef et al., 2014).

ISH for two other hair cell synapse‐associated genes from the paddlefish lateral line organ‐enriched gene set, *Apba1* and *Rab3*, was successful and revealed expression in ampullary organs as well as neuromasts. *Apba1*, encoding the neuronal adaptor protein Apba1 (amyloid‐beta A4 precursor protein‐binding family A member 1; also known as X11α and Munc18‐1‐interacting protein 1, Mint1), was recently identified as a pan‐hair cell marker from mouse utricle scRNA‐seq data (Jan et al., 2021). Although its precise role in hair cells has not been studied, Apba1 (X11α/Mint1) forms part of a presynaptic protein complex with calcium/calmodulin (CaM)‐dependent serine protein kinase (CASK) and neurexin (see, for example, LaConte et al., 2016; Wu et al., 2020). In late‐larval paddlefish, this gene (originally uncharacterised Locus_5717) was 11.4‐fold enriched in operculum versus fin tissue (Modrell, Lyne, et al., 2017). In sterlet at Stage 45, *Apba1* was expressed in ampullary organs as well as neuromasts (Figure 2p–r), suggesting that Apba1 is likely to be associated with the ribbon synapse in electroreceptors as well as hair cells.

*Rab3a* (10.3‐fold lateral line organ‐enriched; Modrell, Lyne, et al., 2017) encodes a small GTP‐binding protein, Ras‐related protein Rab3A, which is a marker for synaptic vesicles in hair cells and is required for their acidification, an essential step in loading neurotransmitter (David et al., 2025; Einhorn et al., 2012; Uthaiah & Hudspeth, 2010). In sterlet at Stage 45, *Rab3a* was expressed in ampullary organs as well as neuromasts (Figure 2s–u), suggesting that Rab3a likely plays a similar role in electroreceptors.

### 3.4 *Nrxn3*, encoding a presynaptic cell adhesion molecule, is expressed in sterlet neuromasts and ampullary organs

*Neurexin 3* (*Nrxn3*) was the only neurexin gene in the late‐larval paddlefish lateral line organ‐enriched dataset (3.1‐fold enriched in operculum versus fin tissue; Modrell, Lyne, et al., 2017). Neurexins are presynaptic cell adhesion molecules that function as synaptic organisers, forming trans‐synaptic bridges with multiple ligands including secreted cerebellins and post‐synaptic transmembrane neuroligins (reviewed by Gomez et al., 2021; Südhof, 2023). RNA‐seq studies have identified *Nrxn3* expression in embryonic and post‐natal hair cells in mouse (Elkon et al., 2015; Sadler et al., 2020; Scheffer et al., 2015) and zebrafish (Lush et al., 2019). A recent study confirmed expression of the long alpha form of both *nrxn3a* and *nrxn3b* in zebrafish hair cells and reported mutant analysis showing that *nrxn3* is required for the maturation of ribbon synapses in zebrafish neuromasts, and in the inner ear of zebrafish and mouse (Jukic et al., 2024).

**FIGURE 4:**
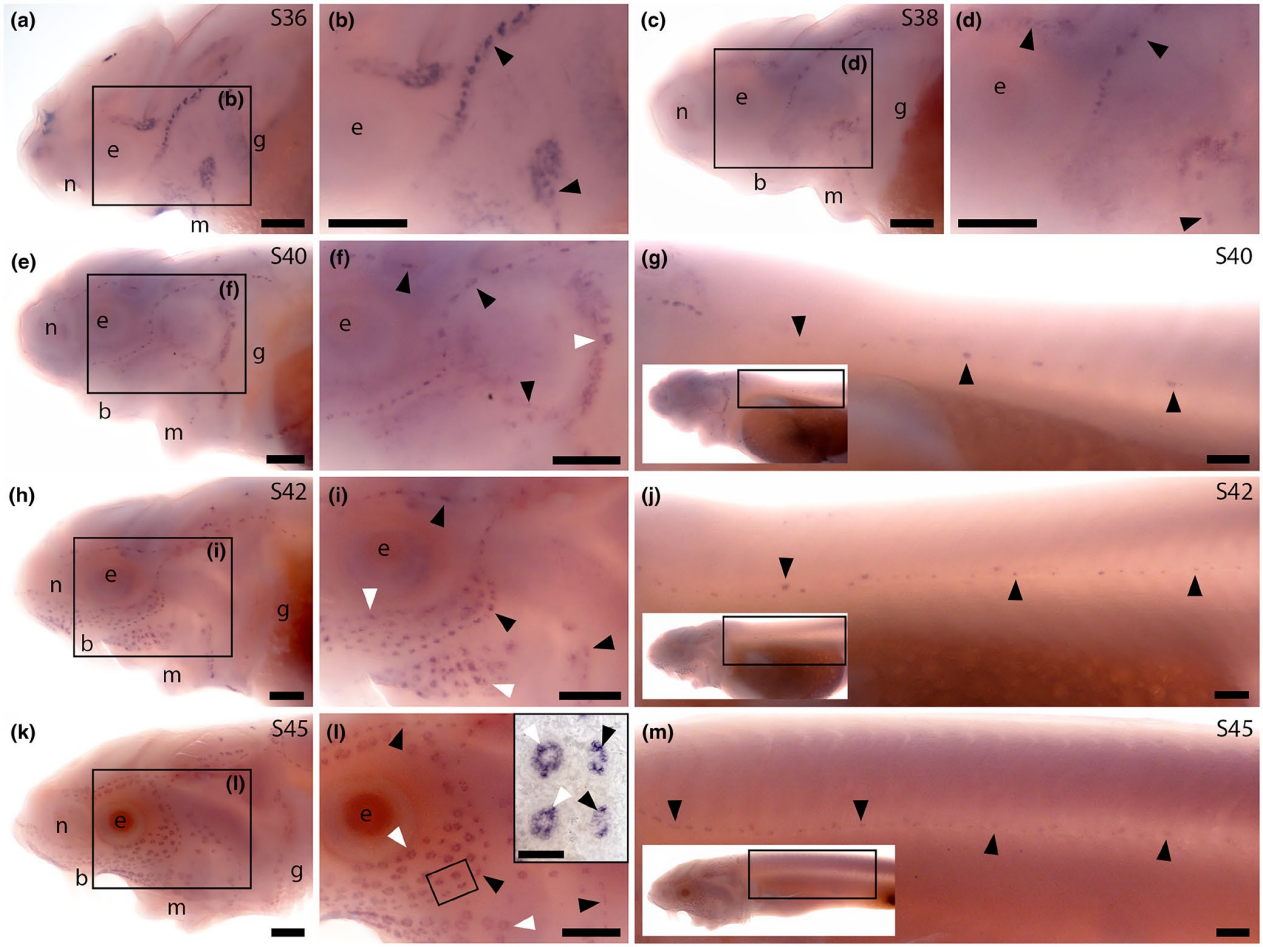
Sterlet *Nrxn3* is expressed in neuromasts and ampullary organs. In situ hybridisation in sterlet for *Nrxn3*. Black arrowheads indicate examples of neuromasts; white arrowheads indicate examples of ampullary organs. For images of the trunk, boxes on low‐power insets delineate the regions shown. (a–d) *Nrxn3* expression is seen in neuromasts on the head from Stage 36 (a, b), persisting at Stage 38 (c, d). (e–g) At Stage 40, *Nrxn3* expression is maintained in cranial neuromasts and is also now seen in ampullary organs (e,f) and neuromasts on the trunk (g). (h–j) At Stage 42, *Nrxn3* expression persists in cranial neuromasts and ampullary organs (h,i) and in neuromasts on the trunk (j). (k–m) At Stage 45, *Nrxn3* expression is maintained in neuromasts and ampullary organs on the head (k, l; inset in l shows a skin mount of ampullary organs from the boxed region) and neuromasts on the trunk (m). Abbreviations: b, barbel; e, eye; g, gill filaments; m, mouth; n, naris; S, stage. Scale bar: 250 μm.

We designed a sterlet *Nrxn3* riboprobe that targets the C‐terminal protein‐coding exon. This exon is included in many of the predicted transcript variants encoding long alpha protein isoforms and all of the predicted *Nrxn3* transcript variants encoding short beta protein isoforms. (In the other predicted transcript variants encoding long alpha protein isoforms, part of this exon is missing.) Hence, we expect our riboprobe to target transcripts encoding both alpha and beta isoforms. At Stage 36, *Nrxn3* was expressed in neuromasts on the head (Figure 4a,b). Expression in cranial neuromasts was maintained at Stage 38 (Figure 4c,d) and at Stage 40, when expression was also seen in some ampullary organs and in neuromasts on the trunk (Figure 4e–g). *Nrxn3* expression persisted in neuromasts and ampullary organs at Stage 42 (Figure 4h–j) and at Stage 45 (Figure 4k–m; the onset of independent feeding; 14 days post fertilisation; Zeiske et al., 2003). The spatiotemporal pattern of sterlet *Nrxn3* expression correlates with the appearance of differentiated hair cells and electroreceptors (Minařík et al., 2024; Figure 1n–q), suggesting it is most likely expressed in electroreceptors as well as hair cells. Given that Nrxn3 is required for the maturation of ribbon synapses in zebrafish and mouse hair cells (Jukic et al., 2024), our sterlet data suggest that Nrxn3 may also be important for the maturation of electroreceptor ribbon synapses.

### 3.5 *Cbln18*, encoding a secreted trans‐synaptic scaffolding glycoprotein, is expressed in sterlet neuromasts but not ampullary organs

*Cerebellin 18* (*Cbln18*) was one of the most highly lateral line organ‐enriched genes (and the only cerebellin gene) identified in our late‐larval paddlefish differential RNA‐seq screen (33.3‐fold enriched in operculum versus fin tissue; Modrell, Lyne, et al., 2017). Cerebellins are secreted scaffolding glycoproteins that bridge synapses by forming tripartite complexes with presynaptic neurexins (specifically, the long alpha forms of Nrxn1‐3) and post‐synaptic proteins, such as the delta glutamate receptors GluD1 and GluD2 (for Cbln1‐3) or the netrin receptors neogenin‐1 and DCC (for Cbln4) (Ferrer‐Ferrer & Dityatev, 2018; Südhof, 2023).

**FIGURE 5:**
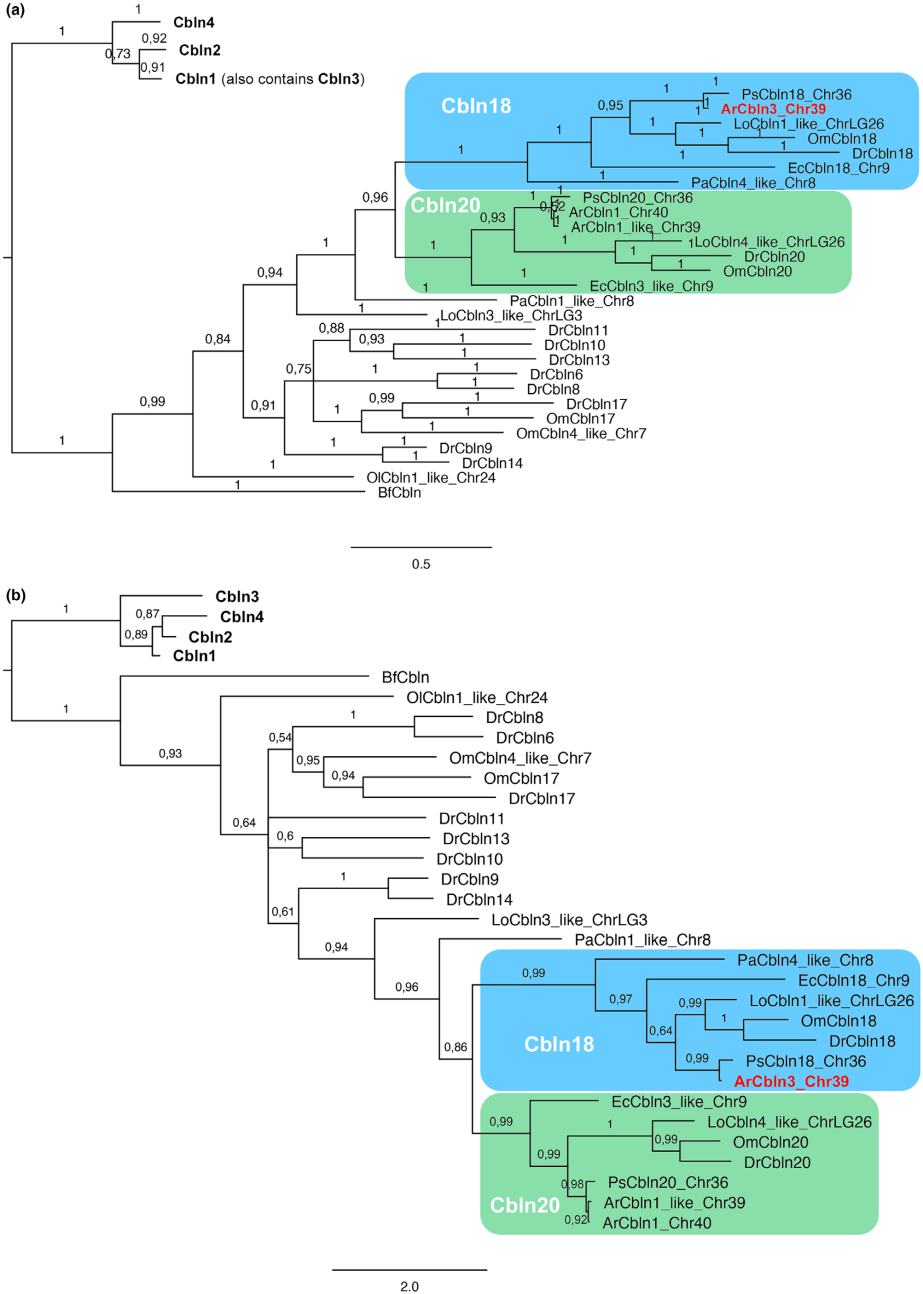
Phylogenetic analysis confirms that the sterlet ortholog of the lateral line organ‐enriched paddlefish *cerebellin* transcript encodes Cbln18. (a, b) Selected portions of phylogenetic trees generated by Bayesian phylogenetic analysis of cerebellin family amino acid sequences using (a) MrBayes v.3.2.7 (Ronquist et al., 2012) and (b) PhyloBayes MPI v1.8c (Lartillot et al., 2013). The complete trees are provided in Figure S1 and Figure S2, respectively. Sequence names reflect the reference‐genome annotation and show the chromosomal (Chr) location of the gene if multiple *cerebellin* genes in that species share the same or similar annotation. Maximum support for the Cbln18 clade in both trees shows that the lateral line‐enriched paddlefish *cerebellin* transcript (Modrell, Lyne, et al., 2017) and its sterlet ortholog (highlighted in bold red font) encode Cbln18 and have been mis‐annotated as *Cbln3* in the respective reference genomes (paddlefish GCF_017654505.1; sterlet GCF_902713425.1). Both trees also show that the sterlet *Cbln20* ohnologs have been mis‐annotated in the reference genome as *Cbln1‐like* (chromosome 39) and *Cbln1* (chromosome 40). GenBank accession numbers for the sequences used (104 from vertebrates plus the single amphioxus cerebellin sequence) are given in Table S2. Species abbreviations: Ar, *Acipenser ruthenus*; Am, *Alligator mississippiensis*; Bf, *Branchiostoma floridae*; Cl, *Canis lupus*; Cm, *Callorhinchus milii*; Dr, *Danio rerio*; Ec, *Erpetoichthys calabaricus*; Gg, *Gallus gallus*; Hs, *Homo sapiens*; Lo, *Lepisosteus oculatus*; Mm, *Mus musculus*; Ol, *Oryzias latipes*; Om, *Oncorhynchus mykiss*; Pa, *Protopterus annectens*; Ps, *Polyodon spathula*; Sc, *Scyliorhinus canicula*; Xt, *Xenopus tropicalis*.

Phylogenetic analysis of vertebrate cerebellin proteins confirmed the identity of the lateral line organ‐enriched paddlefish *cerebellin* transcript (Modrell, Lyne, et al., 2017) and its sterlet ortholog as *Cbln18* (Figure 5a,b; Figures S1 and S2; Table S2). The chondrostean lineage underwent a whole‐genome duplication (Du et al., 2020; Redmond et al., 2023) but only a single *Cbln18* ohnolog (on chromosome 39) was identified in the reference genome (GCF_902713425.1), where it has been misannotated as *Cbln3* (Figure 2; Figures S1 and S2). The paddlefish *Cbln18* gene has also been misannotated as *Cbln3* in the reference genome (GCF_017654505.1) (Figure 2a,b; Figures S1 and S2). However, *Cbln3* seems to be mammal‐specific: no Cbln proteins from non‐mammalian vertebrates cluster with Cbln3 (Figures S1 and S2).

**FIGURE 6:**
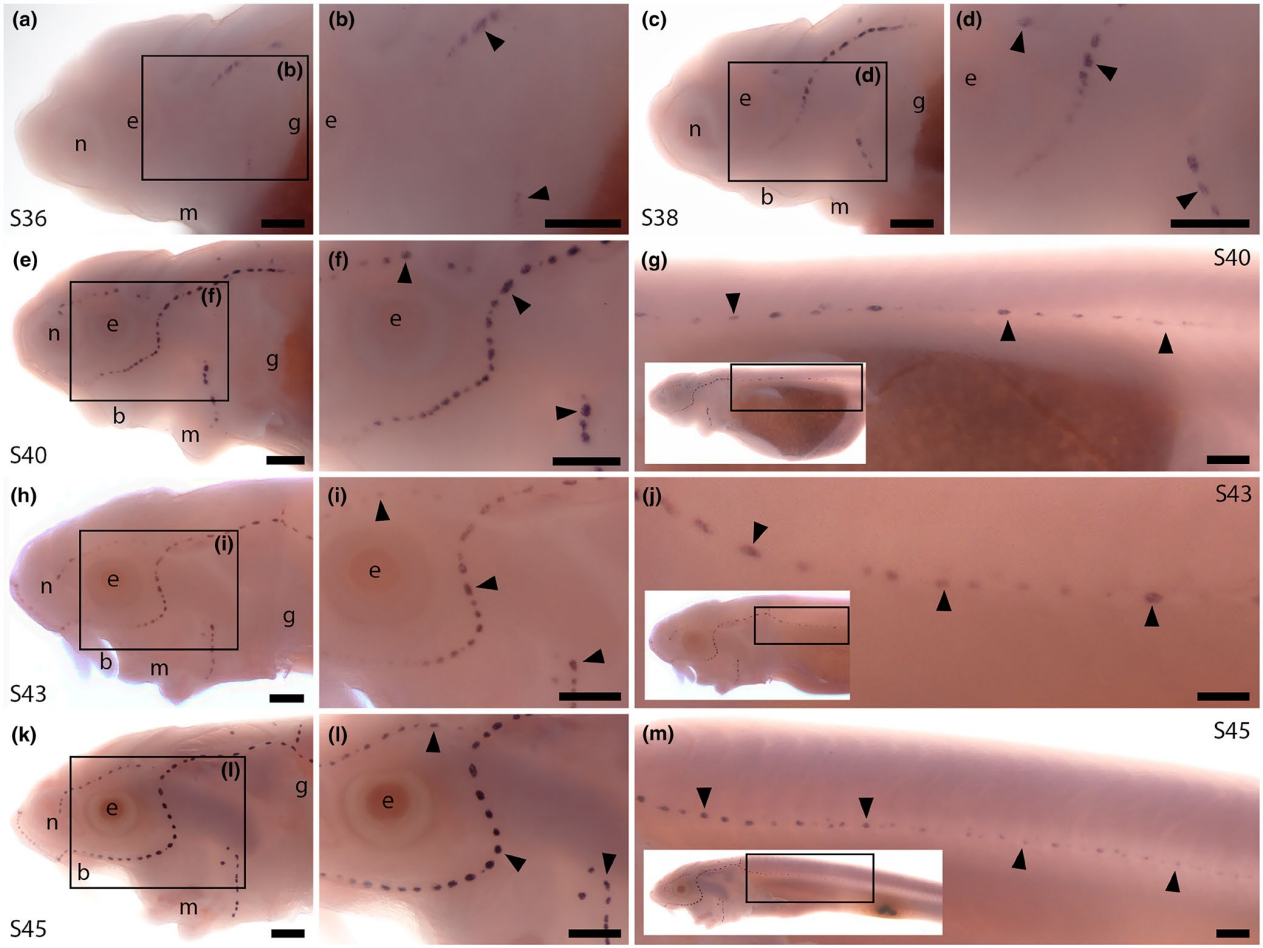
Sterlet *Cbln18* is expressed in neuromasts but not ampullary organs. In situ hybridisation in sterlet for *Cbln18*. Black arrowheads indicate examples of neuromasts. For images of the trunk, boxes on low‐power insets delineate the regions shown. (a–d) *Cbln18* expression is seen in neuromasts on the head at Stage 36 (a, b), persisting at Stage 38 (c, d). (e–g) At Stage 40, expression is maintained in cranial neuromasts (e, f) and is also visible in trunk neuromasts (g). (h–m) At Stages 42 (h–j) and 45 (k–m), *Cbln18* expression persists in neuromasts on the head (h, i, k, l) and trunk (j, m). Abbreviations: b, barbel; e, eye; g, gill filaments; m, mouth; n, naris; S, stage. Scale bar: 250 μm.

No information has been published about the expression or function of zebrafish *cbln18*. ScRNA‐seq data (as‐yet unvalidated by direct expression studies) show expression of *cbln18* and the related gene *cbln20* (Figures S1 and S2) in the central support cells of neuromasts, but not in mature hair cells (Lush et al., 2019; Sur et al., 2023). Central support cells surround hair cells and also act as hair cell progenitors during homeostasis and regeneration (Lush et al., 2019). ISH in sterlet showed strong *Cbln18* expression in neuromasts on the head at Stage 36 and Stage 38 (Figure 6a–d) and on both the trunk and head from Stage 40 until Stage 45 (the latest stage examined) (Figure 6e–m). No expression was detected at any stage in ampullary organs (in which electroreceptors start to differentiate from Stages 40 to 41; Minařík et al., 2024). This suggests that Cbln18 may be important specifically for the synapses between hair cells and their afferent axon terminals, but not for electroreceptor synapses.

### 3.6 *Pcp4*, encoding calmodulin‐regulator protein PCP4 (PEP‐19), is expressed in sterlet neuromasts and ampullary organs

*Purkinje cell protein 4* (*Pcp4*) was 13.2‐fold lateral line organ‐enriched in late‐larval paddlefish (Modrell, Lyne, et al., 2017). *Pcp4* encodes calmodulin‐regulator protein PCP4 (also known as PEP‐19), which binds to the intracellular Ca^2+^ sensor calmodulin in a Ca^2+^‐independent manner and can suppress calmodulin‐dependent signalling, preventing cellular Ca^2+^ overload and protecting against glutamate‐induced excitotoxicity in neurons (Kanazawa et al., 2008; Slemmon et al., 1996; Slemmon et al., 2000; Wang et al., 2013). *Pcp4* is expressed in mouse inner‐ear hair cells (Burns et al., 2015; Cai et al., 2015; Thomas et al., 2003) but its role in hair cells is unknown.

**FIGURE 7:**
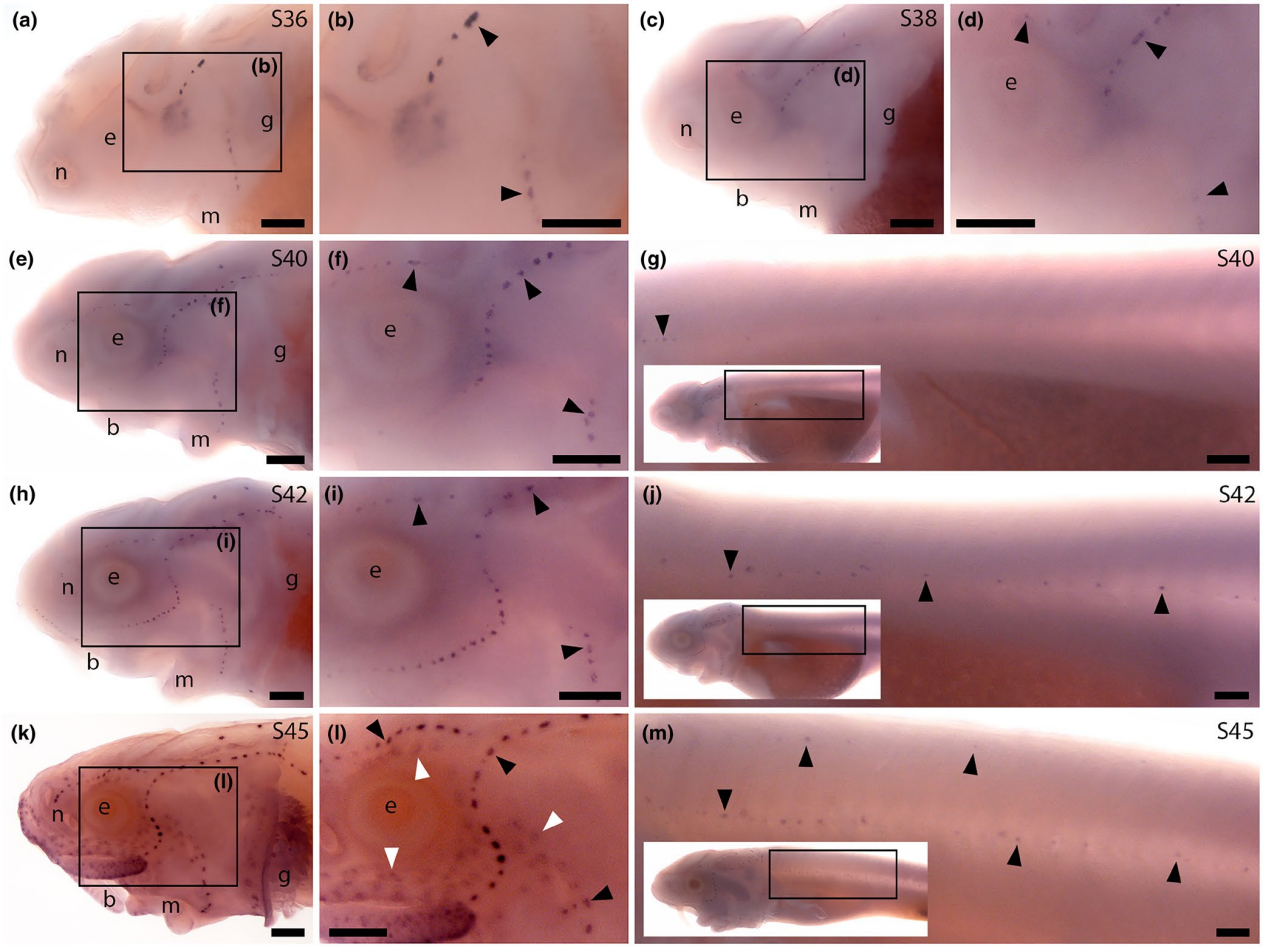
Sterlet *Pcp4* is expressed in neuromasts and ampullary organs. In situ hybridisation in sterlet for *Pcp4*. Black arrowheads indicate examples of neuromasts; white arrowheads indicate examples of ampullary organs. For images of the trunk, boxes on low‐power insets delineate the location of the trunk regions shown. (a–d) *Pcp4* expression is seen in neuromasts on the head at Stage 36 (a, b) and Stage 38 (c, d). (e–j) At Stage 40 (e–g) and Stage 42 (h–j), *Pcp4* expression is maintained in neuromasts on the head and is also visible in neuromasts on the trunk. (k–m) At Stage 45, neuromast expression is maintained on the head and weaker expression in ampullary organs also appears (k, l). On the trunk, expression continues in neuromasts (m). Abbreviations: b, barbel; e, eye; g, gill filaments; m, mouth; n, naris; S, stage. Scale bar: 250 μm.

Preliminary ISH data from late‐larval paddlefish (not shown) suggested *Pcp4* was expressed in both neuromasts and ampullary organs. In sterlet, ISH revealed strong *Pcp4* expression in neuromasts on the head already at Stage 36 (shortly after the first differentiated hair cells are seen at Stage 35; Minařík et al., 2024) and Stage 38 (Figure 7a–d), and on the head and trunk at Stage 40, Stage 42 and Stage 45 (Figure 7e–m). At Stage 45, weaker *Pcp4* expression was also seen in ampullary organs (Figure 7k,l), suggesting PCP4 is important for the function of electroreceptors, as well as hair cells. However, differentiated electroreceptors are first seen from Stages 40 to 41 (Minařík et al., 2024). The lack of detectable *Pcp4* expression at Stage 42, versus strong *Pcp4* expression in neuromasts at Stage 36, shortly after the first differentiated hair cells are seen (Minařík et al., 2024), suggests that the function of PCP4 may differ in ampullary organs and neuromasts.

### 3.7 *Syt14*, encoding a calcium‐independent synaptotagmin, is expressed in sterlet neuromasts and ampullary organs

*Synaptotagmin 14* (*Syt14*) was 12.0‐fold lateral line organ‐enriched in late‐larval paddlefish (Modrell, Lyne, et al., 2017). Synaptotagmins are transmembrane proteins with two cytoplasmic Ca^2+^‐binding C2 domains (Wolfes & Dean, 2020). *Syt14* is expressed in embryonic and postnatal inner ear hair cells in mouse (Scheffer et al., 2015) and in utricular (vestibular) hair cells in chicken (Scheibinger et al., 2022). In zebrafish, scRNA‐seq data show expression of *syt14b* in hair cells in the inner ear and neuromasts (Sur et al., 2023). Many synaptotagmins are important for the regulation of calcium‐dependent membrane fusion events, with some acting as fast calcium sensors for synaptic vesicle exocytosis (Wolfes & Dean, 2020). In contrast, Syt14 is calcium‐independent and its precise function is unknown (Fukuda, 2003; Wolfes & Dean, 2020).

**FIGURE 8:**
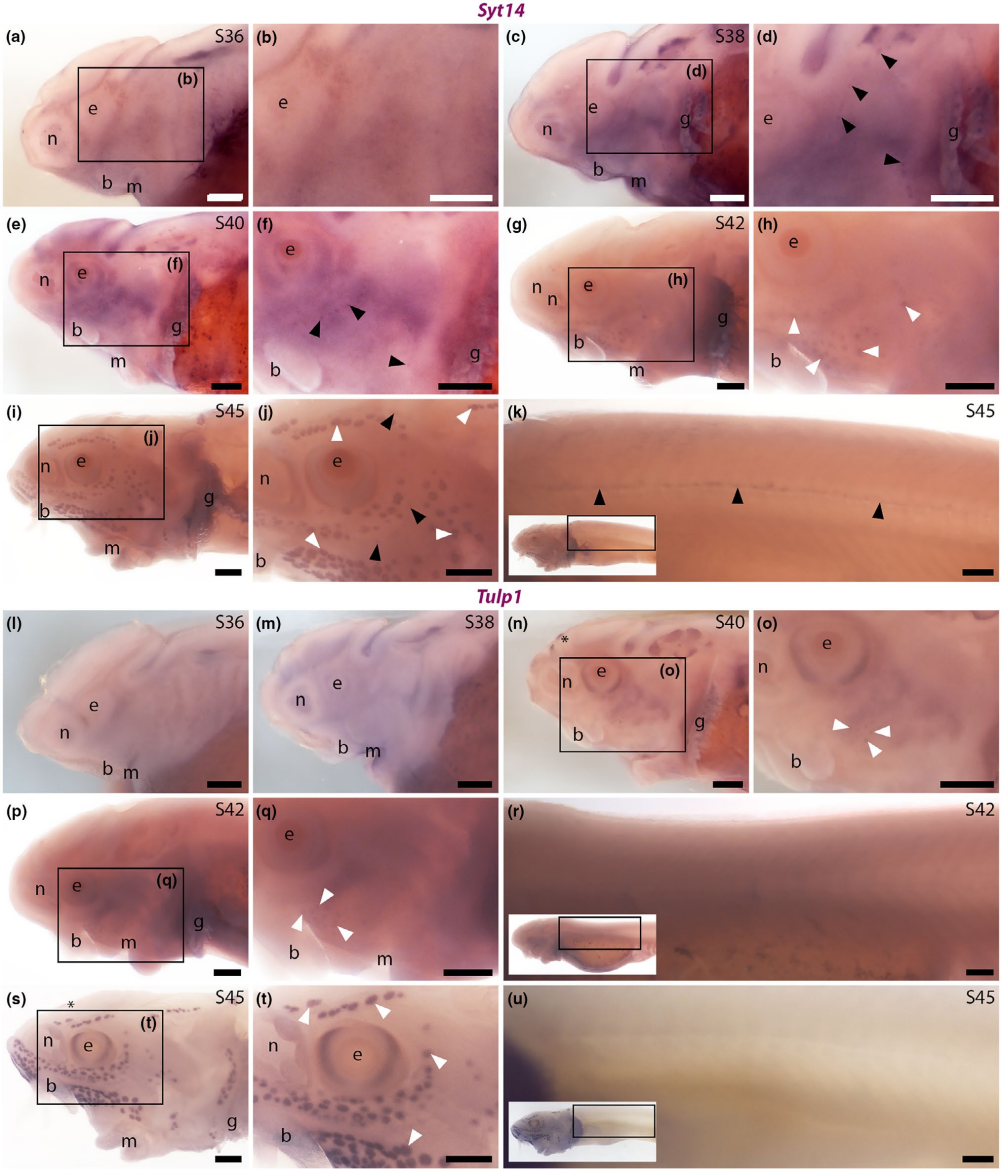
Sterlet neuromasts and ampullary organs express *Syt14*, but only ampullary organs express *Tulp1*. In situ hybridisation in sterlet. Black arrowheads indicate examples of neuromasts; white arrowheads indicate examples of ampullary organs. For images of the trunk, boxes on low‐power insets delineate the location of the trunk regions shown. (a, b) No specific *Syt14* expression is seen at Stage 36. (c–f) At Stage 38 (c, d) and Stage 40 (e, f), weak *Syt14* expression is observed in cranial neuromasts. (g, h) At Stage 42, weak *Syt14* expression is seen in ampullary organs. (i–k) At Stage 45, *Syt14* expression is maintained in ampullary organs (h, i) and weaker expression is seen in neuromasts on the head (i, j) and trunk (k). (l, m) No specific *Tulp1* expression is seen at either Stage 36 (l) or Stage 38 (m). (n, o) At Stage 40, *Tulp1* expression is observed in the eye, a midline patch between the eyes that is most likely the epiphysis (asterisk), and weakly in ampullary organs. No expression is seen in neuromasts. (p–r) At Stage 42, *Tulp1* expression continues in ampullary organs but not in neuromasts, either on the head (p, q) or trunk (r). (s–u) At Stage 45, *Tulp1* expression is maintained in the eye, weakly in the presumed epiphysis (asterisk), and in ampullary organs (s, t). No expression is seen in neuromasts, either on the head (s, t) or trunk (u). Abbreviations: b, barbel; e, eye; g, gill filaments; m, mouth; n, naris; S, stage. Scale bar: 250 μm.

In sterlet, ISH for *Syt14* revealed weak expression in developing neuromasts at Stage 38 and Stage 40 (Figure 8a–f) and in ampullary organs at Stage 42 (Figure 8g,h). At Stage 45, *Syt14* expression was seen in ampullary organs and, more weakly, in neuromasts on both head and trunk (Figure 8i–k). These data suggest that the (unknown) function of Syt14 in hair cells is likely conserved in electroreceptors.

### 3.8 *Tulp1*, encoding tubby‐related protein 1, is expressed in sterlet ampullary organs but not neuromasts

*TUB like protein 1* (*Tulp1*) was 2.4‐fold lateral line organ‐enriched in late‐larval paddlefish (Modrell, Lyne, et al., 2017). Tulp1 is a member of the tubby family of proteins, characterised by a conserved C‐terminal ‘tubby’ domain that binds phosphoinositide 4,5‐bisphosphate (PIP2), linking the protein to the membrane (Mukhopadhyay & Jackson, 2011). In mice, Tulp1 is restricted to the retina, where it interacts with Ribeye (the main structural component of the presynaptic ribbon) and is required for the normal development and function of the photoreceptor ribbon synapse (Grossman et al., 2009; Wahl et al., 2016). In zebrafish, scRNA‐seq data show *tulp1a* and *tulp1b* expression in photoreceptors and photoreceptor precursors but not in lateral line or inner ear hair cells (Sur et al., 2023).

ISH showed no detectable expression of sterlet *Tulp1* at Stage 36 or Stage 38 (Figure 8l,m). At Stage 40 and Stage 42, *Tulp1* expression was seen in the eye and, weakly, in ampullary organs, but not in neuromasts (Figure 8n–r). At Stage 45, *Tulp1* expression was maintained in the eye and in ampullary organs (Figure 8s,t). No *Tulp1* expression was seen in neuromasts, either on the head (Figure 8s,t) or trunk (Figure 8u). Thus, Tulp1 seems to be ampullary organ‐specific within the developing lateral line system. Given the importance of Tulp1 for the development and function of the photoreceptor ribbon synapse (Grossman et al., 2009; Wahl et al., 2016), these data suggest that Tulp1 may be important specifically for the ribbon synapse of electroreceptors, but not hair cells.

### 3.9 *Dscaml1*, encoding cell adhesion molecule DSCAML1, is expressed in sterlet neuromasts and ampullary organs

*Down's syndrome cell adhesion molecule‐like 1* (*Dscaml1*) was 25.3‐fold lateral line organ‐enriched in late‐larval paddlefish (Modrell, Lyne, et al., 2017). *Dscaml1* encodes cell adhesion molecule DSCAML1, a homophilic transmembrane cell adhesion molecule of the immunoglobulin superfamily (Agarwala et al., 2001). In the mouse retina, *Dscaml1* is expressed in rod photoreceptors, rod bipolar cells (which also form ribbon synapses, like photoreceptors) and AII amacrine cells (Fuerst et al., 2009). In *Dscaml1*‐null retinas, ribbon synapses between rod bipolar cells and AII amacrine cells are functional but seem to be immature, with fewer ribbons, some floating (detached from the presynaptic membrane), significantly more synaptic vesicles and a slower decay of the synaptic current (Fuerst et al., 2009; also see Garrett et al., 2016). In zebrafish, scRNA‐seq data show *Dscaml1* expression in hair cells in neuromasts and the inner ear, as well as in retinal bipolar cells and amacrine cells (Sur et al., 2023).

**FIGURE 9:**
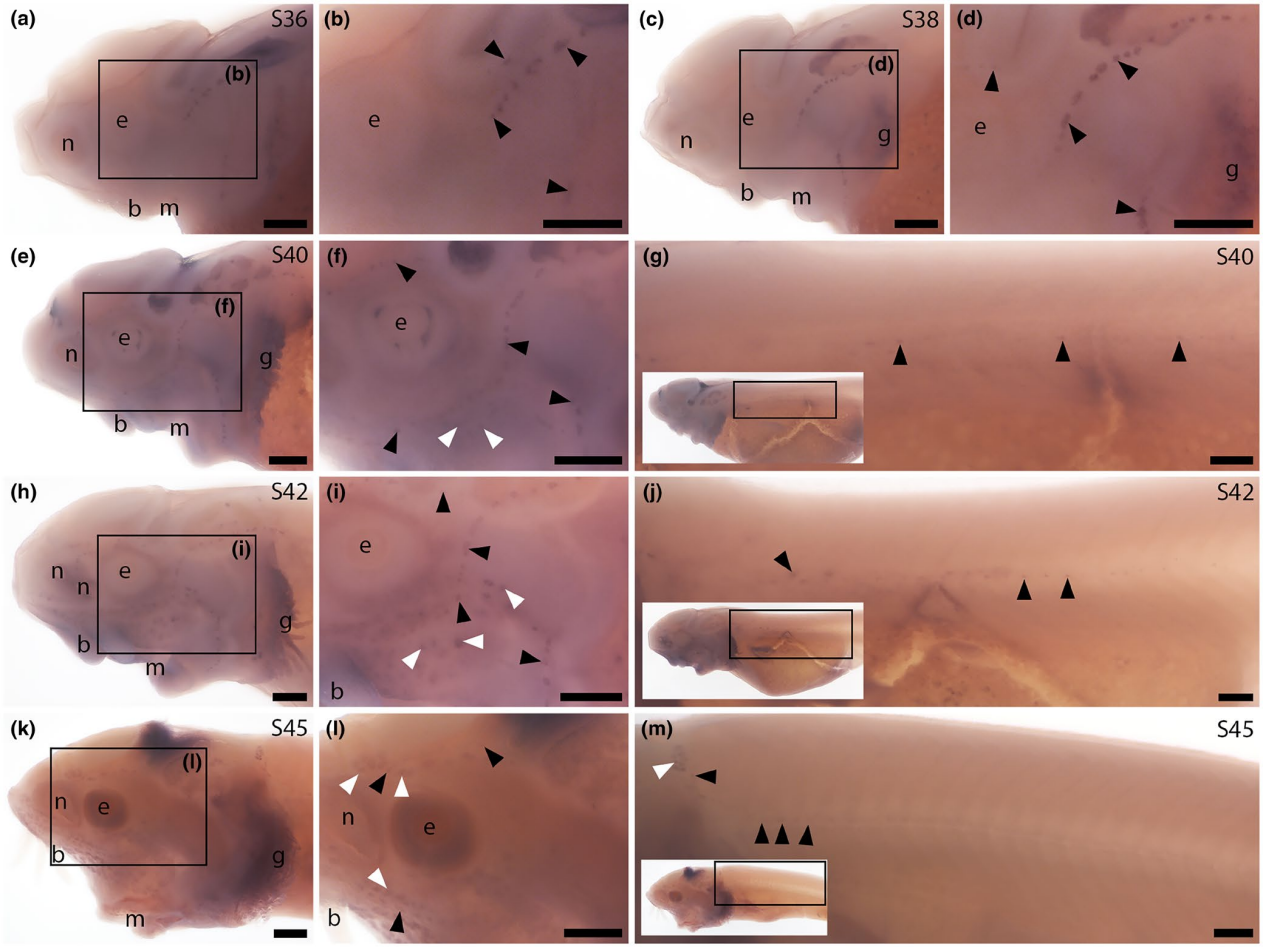
Sterlet *Dscaml1* is expressed in neuromasts and ampullary organs. In situ hybridisation in sterlet for *Dscaml1*. Black arrowheads indicate examples of neuromasts; white arrowheads indicate examples of ampullary organs. For images of the trunk, boxes on low‐power insets delineate the location of the trunk regions shown. (a–d) *Dscaml1*expression is seen in neuromasts on the head at Stage 36 (a, b) and Stage 38 (c, d). (e–j) At Stage 40 (e–g) and Stage 42 (h–j), *Dscaml1* expression continues in neuromasts on the head and is also seen in neuromasts on the trunk, as well as ampullary organs. (k–m) At Stage 45, *Dscaml1* expression is maintained in ampullary organs (k, l) but neuromast expression seems weaker, especially on the trunk where it is only detectable rostrally (m). Abbreviations: b, barbel; e, eye; g, gill filaments; m, mouth; n, naris; S, stage. Scale bar: 250 μm.

In sterlet, ISH for *Dscaml1* revealed strong expression in neuromasts on the head at Stage 36 and Stage 38 (Figure 9a–d), and on the head and trunk at Stage 40, Stage 42 and Stage 45 (Figure 9e–m), although at Stage 45, expression seemed to be fading. At Stage 40, Stage 42 and Stage 45, *Dscaml1* expression was also seen in ampullary organs (Figure 9e–m).

We also tested expression in sterlet of the related gene *Dscam*, which was 3.5‐fold enriched in late‐larval paddlefish (Modrell, Lyne, et al., 2017). However, no specific *Dscam* expression was seen in developing lateral line organs (data not shown).

## 4 DISCUSSION

We studied the developmental expression of a range of synapse‐related genes selected from a paddlefish lateral line organ‐enriched transcriptome (Modrell, Lyne, et al., 2017) in a related chondrostean fish, the sterlet sturgeon. Their various expression patterns provide insights into the structure and potential physiology of ribbon synapses in electroreceptors, at least in non‐teleost bony ray‐finned fish, identifying both conserved aspects and molecular differences with ribbon synapses in hair cells.

### 4.1 Expression of *Slc17a8* and *Slc1a3* in ampullary organs supports electroreceptor synapses being glutamatergic

Hair cell ribbon synapses are glutamatergic (Johnson et al., 2019; Moser et al., 2020), but the excitatory neurotransmitter at electroreceptor ribbon synapses has not been identified (Bodznick & Montgomery, 2005; Leitch & Julius, 2019). We previously reported the expression in late‐larval paddlefish neuromasts and ampullary organs of *Slc17a8*, encoding vGlut3 (Modrell, Lyne, et al., 2017), which is required to refill synaptic vesicles at hair cell (but not photoreceptor) ribbon synapses (see Johnson et al., 2019; Moser et al., 2020). Here, we confirmed that *Slc17a8* is expressed in late‐larval neuromasts and ampullary organs in sterlet (as expected, given that both are chondrostean fish). Further, we found that larval sterlet neuromasts and ampullary organs also express *Slc1a3*, encoding EAAT1 (GLAST). This transporter is required for supporting cells of the mammalian inner ear to take up glutamate from hair cell ribbon synapses (Chen et al., 2010; Glowatzki et al., 2006). In zebrafish neuromasts, *Slc1a3* is expressed in central and polar support cells and mantle cells (Lush et al., 2019). Shared expression of both *Slc1a3* (EAAT1/GLAST) and *Slc17a8* (vGlut3) in ampullary organs and neuromasts strengthens the proposal that ribbon synapses are glutamatergic in electroreceptors as well as hair cells.

### 4.2 Expression of *Nrxn3* and *Cbln18* sheds light on the structure of ribbon synapses in hair cells versus electroreceptors

The presynaptic cell adhesion molecule Nrxn3 was recently shown to be required for the maturation of hair‐cell ribbon synapses in zebrafish and mouse (Jukic et al., 2024). We identified shared expression of *Nrxn3* in sterlet neuromasts and ampullary organs, suggesting that Nrxn3 may play the same role at electroreceptor ribbon synapses. The specific binding partners of Nrxn3 in hair cells and electroreceptors are unknown. Neurexins commonly partner directly with neuroligins in the post‐synaptic membrane: for example, in the mouse cochlea, Nrxn1 and Nlgn3 are both important for the maturation of hair‐cell ribbon synapses (Ramirez et al., 2022). The paddlefish lateral line organ‐enriched gene set (Modrell, Lyne, et al., 2017) did not include any neuroligin genes, but this is not surprising as the mRNA would be expressed in afferent lateral line neurons. However, our gene expression data suggest that one synaptic partner for Nrxn3 in sterlet hair cells, but not electroreceptors, is likely to be Cbln18, a member of the cerebellin family of secreted scaffolding glycoproteins (Ferrer‐Ferrer & Dityatev, 2018; Südhof, 2023). Our phylogenetic analysis showed that *Cbln18* is one of a group of cerebellin genes not found in mammals. *Cbln18* was strongly expressed in sterlet neuromasts throughout development, but not seen at any stage in ampullary organs. At the hair cell ribbon synapse, pre‐synaptic Nrxn3 and secreted Cbln18 likely form a tripartite complex with glutamate receptors on the post‐synaptic membrane (Ferrer‐Ferrer & Dityatev, 2018; Gomez et al., 2021; Südhof, 2023). *Cbln18* was the only cerebellin gene in the paddlefish lateral line organ‐enriched gene set (Modrell, Lyne, et al., 2017), but this dataset is not exhaustive and it is possible that a different cerebellin gene is expressed in electroreceptors. Taken together, our data suggest that ribbon synapses in hair cells and electroreceptors include presynaptic Nrxn3, but only hair‐cell ribbon synapses include secreted Cbln18.

### 4.3 Expression of *Syt14*, *Tulp1* and *Dscaml1* gives further insight into ribbon synapses in hair cells versus electroreceptors

Another synapse‐associated gene with shared expression in developing sterlet neuromasts and ampullary organs was *Syt14*, encoding a calcium‐independent member of the synaptotagmin family (Wolfes & Dean, 2020) that is expressed in mouse and chicken hair cells (Scheffer et al., 2015; Scheibinger et al., 2022). In zebrafish, scRNA‐seq data also show *Syt14b* expression in otic and lateral line hair cells (Sur et al., 2023). The better‐studied family members Syt1, Syt2, Syt7 and Syt9 are transmembrane synaptic vesicle proteins that act as calcium sensors and regulate synaptic vesicle exocytosis at synapses in the central nervous system (Wolfes & Dean, 2020). However, at mature hair cell ribbon synapses, otoferlin is the calcium sensor that controls synaptic vesicle exocytosis, rather than synaptotagmins (reviewed by Johnson et al., 2019; Moser et al., 2020). Furthermore, Syt14 is calcium‐independent (Fukuda, 2003). Syt14 is expressed in cerebellar Purkinje cells and human *SYT14* mutation is associated with spinocerebellar ataxia (Doi et al., 2011), but its precise function is unknown. Another calcium‐independent synaptotagmin, Syt4, both inhibits vesicle exocytosis and slows endocytosis (reviewed by Wolfes & Dean, 2020). Given that we observed *Syt14* expression in both ampullary organs and neuromasts, it is possible that Syt14 could similarly be involved in regulating exocytosis and/or endocytosis at both electroreceptor and hair cell ribbon synapses. This is of course only a speculative hypothesis in the absence of physiological data.

*Dscaml1*, encoding the homophilic cell adhesion molecule DSCAML1 (Agarwala et al., 2001), was also expressed in developing neuromasts and ampullary organs in sterlet. (The related gene *Dscam* did not show specific expression in developing lateral line organs.) Data from *Dscaml1*‐null mouse retinas suggested that *Dscaml1* may be required for the final maturation of ribbon synapses between Dscaml1‐expressing rod bipolar cells and AII amacrine cells (Fuerst et al., 2009; Garrett et al., 2016). Although these ribbon synapses were functional in *Dscaml1*‐null retinas, fewer ribbons formed; some ribbons were not associated with the presynaptic membrane (‘floating’) and significantly more synaptic vesicles were present, suggesting a potential defect in exocytosis (Fuerst et al., 2009). These features were described as suggestive of immature synapses (Garrett et al., 2016). *Dscaml1* expression has not been published in hair cells, to our knowledge; in zebrafish, *Dscaml1* expression has only been examined in the retina (Galicia et al., 2018; Ma et al., 2020). However, scRNA‐seq data suggest that inner ear and neuromast hair cells indeed express *Dscaml1* (Sur et al., 2023). It is possible that Dscaml1 might play a similar role in the maturation of ribbon synapses between neuromast hair cells/electroreceptors and their afferent neurons, assuming the latter also express *Dscaml1*.

In contrast to the shared expression in neuromasts and ampullary organs of *Syt14* and *Dscaml1*, we saw differential expression of a gene that is important for regulating endocytosis at the photoreceptor ribbon synapse, *Tulp1* (Wahl et al., 2016). This gene, which encodes tubby‐related protein 1, is mutated in a subset of human patients with retinitis pigmentosa, in which photoreceptors degenerate (reviewed by Frederick & Zenisek, 2023). In mice, Tulp1 is restricted to the retina, where it is required for endocytosis in the periactive zone of the photoreceptor ribbon synapse (Wahl et al., 2016). Such endocytosis is essential for the continued release of synaptic vesicles at the photoreceptor synapse (Wen et al., 2018). Sterlet *Tulp1* was expressed in developing ampullary organs, but not neuromasts. (Similarly, zebrafish scRNA‐seq data show no expression of *tulp1a* or *tulp1b* in otic or lateral line cells; Sur et al., 2023.) Expression was also seen in a dorsal midline patch likely to be the epiphysis (pineal gland), as reported for both *tulp1a* and *tulp1b* in zebrafish (Jia et al., 2022). Pinealocytes also have ribbon synapses (Moser et al., 2020). Hence, it is possible that Tulp1 could be important for vesicle recycling at the ribbon synapse of electroreceptors and pinealocytes, as it is in photoreceptor ribbon synapses (Frederick & Zenisek, 2023).

*Tulp1* is also required for the normal development of photoreceptor ribbon synapses (Grossman et al., 2009). Tulp1 in photoreceptors interacts with Ribeye (Ebke et al., 2021), the major constituent of the presynaptic ribbon in all ribbon synapses, including in hair cells (Moser et al., 2020; Voorn & Vogl, 2020) and most likely also electroreceptors, given that paddlefish ampullary organs express the Ribeye‐specific A domain of *Ctbp2*, encoding Ribeye (Modrell, Lyne, et al., 2017). In *Tulp1*‐null mice, few intact ribbons are found: the ribbon synapse proteins Piccolo and Bassoon fail to associate properly and the dendrites of associated bipolar cells are much shorter than normal, resulting in malformed synapses (Grossman et al., 2009). By analogy, Tulp1 may play multiple roles at the electroreceptor ribbon synapse, ranging from development to regulating endocytosis.

Tulp1 also has non‐synapse‐related roles in photoreceptors. In the inner segment of photoreceptors, Tulp1 binds to Kif3, a subunit of the Kinesin‐2 motor complex involved in ciliary membrane protein trafficking (Ebke et al., 2021). In *tulp1*‐mutant zebrafish, the primary cilium of photoreceptors is significantly shorter than normal and opsins are mis‐localised (Jia et al., 2022). Electroreceptors in most non‐teleost jawed vertebrates also have an apical primary cilium (Baker & Modrell, 2018; Elliott & Fritzsch, 2020; Jørgensen, 2005). Indeed, a recent immunolabelling study in paddlefish and sturgeon (*Scaphirhynchus platorynchus*) showed that plasma membrane Ca^2+^ pumps are localised to the cilium itself, suggesting the cilium is the site of electrotransduction (Russell et al., 2025). Thus, it is possible that Tulp1 in electroreceptors also has non‐synapse‐related roles in trafficking proteins in the ciliary membrane.

### 4.4 Differential onset and levels of *Pcp4* expression suggest potential differences in its role in electroreceptors versus hair cells

Calmodulin‐regulator protein PCP4 (PEP‐19) binds calmodulin in a Ca^2+^‐independent manner through its IQ domain (Slemmon et al., 1996). Calmodulin is an EF‐hand intracellular Ca^2+^ sensor that interacts with and modifies the activity of hundreds of target proteins required for a plethora of cellular functions (Berchtold & Villalobo, 2014; Chin & Means, 2000; Hussey et al., 2023). PCP4 can downregulate Ca^2+^‐calmodulin signalling and protect against glutamate‐mediated excitotoxicity arising from raised levels of intracellular Ca^2+^ (Kanazawa et al., 2008; Slemmon et al., 2000; Wang et al., 2013). Thus, the expression of *Pcp4* that we identified in larval sterlet neuromasts and ampullary organs (also reported in mouse inner‐ear hair cells; Thomas et al., 2003; Burns et al., 2015; Cai et al., 2015) could protect both hair cells and electroreceptors against glutamate‐mediated excitotoxicity.

However, differences in the onset and relative levels of *Pcp4* expression in neuromasts versus ampullary organs may suggest potential differences in the role(s) of Pcp4 in hair cells versus electroreceptors. In neuromasts, *Pcp4* was strongly expressed in a spatiotemporal pattern correlating closely with hair cell differentiation (as previously detected by expression of *Cacna1d*, encoding the pore‐forming subunit of Ca_v_1.3; Minařík et al., 2024). In contrast, the onset of detectable *Pcp4* expression in ampullary organs was much later relative to electroreceptor differentiation (as previously detected by the expression of *Cacna1d* or electroreceptor‐specific *Kcna5*; Minařík et al., 2024). *Pcp4* expression in ampullary organs was also weaker than in neuromasts, as seen by qualitatively comparing the intensity of ISH staining between ampullary organs and neuromasts in the same larvae. It is possible that ampullary organs use a different calmodulin regulator to protect against glutamate‐mediated excitotoxicity and/or that the requirement for PCP4 differs in electroreceptors and hair cells.

One possibility for differing PCP4 requirements in electroreceptors and hair cells may be the importance of calmodulin for the rapid Ca^2+^‐dependent inactivation of L‐type voltage‐gated calcium channels, such as Ca_v_1.3 (Peterson et al., 1999; Zühlke et al., 1999; reviewed by Ames, 2021). After Ca_v_1.3 opens and intracellular Ca^2+^ levels increase, Ca^2+^‐bound calmodulin binds to Ca_v_1.3, inactivating the channel (Ames, 2021). In hair cells, ribbon synapse‐associated Ca_v_1.3 channels open in response to hair cell depolarisation and the Ca^2+^ influx triggers synaptic vesicle exocytosis (Johnson et al., 2019; Moser et al., 2020). In electroreceptors, Ca_v_1.3 channels most likely have the same function at the ribbon synapse, given that *Cacna1d* was the only pore‐forming Ca_v_ channel gene identified in the paddlefish lateral line organ‐enriched gene set (Modrell, Lyne, et al., 2017) and the predominant pore‐forming Ca_v_ channel mRNA in skate and shark ampullary organs (Bellono et al., 2017; Bellono et al., 2018). However, Ca_v_1.3 plays an additional role in electroreceptors: it was identified as the voltage‐sensing calcium channel in the apical membrane of skate and shark electroreceptors (Bellono et al., 2017; Bellono et al., 2018; reviewed by Leitch & Julius, 2019). Electroreceptor function relies on constant activation of calcium‐driven voltage oscillations mediated by low‐threshold Ca_v_1.3 channels in the apical membrane: the calcium influx in turn activates an outward potassium current (mediated by different potassium channels in skate and shark) (Bellono et al., 2017; Bellono et al., 2018; reviewed by Leitch & Julius, 2019). It is possible that the differences in timing and expression levels of the calmodulin‐regulator gene *Pcp4* in sterlet ampullary organs versus neuromasts relate somehow to this additional role of Ca_v_1.3 in the apical membrane of electroreceptors and the importance of Ca^2+^‐calmodulin for rapid Ca^2+^‐dependent inactivation of Ca_v_1.3 (Ames, 2021).

## 5 CONCLUSION

We found that 11 genes involved in synapse development, stability and/or function were also expressed in developing sterlet lateral line organs. Expression in sterlet neuromasts and ampullary organs of both *Slc17a8*, encoding vGlut3 (as expected from paddlefish; Modrell, Lyne, et al., 2017) and *Slc1a3*, encoding the glutamate re‐uptake transporter EAAT1 (GLAST), supports ribbon synapses being glutamatergic in electroreceptors, as well as hair cells. Genes encoding known hair cell synapse‐associated proteins were expressed in ampullary organs as well as neuromasts, supporting molecular conservation: otoferlin (as expected from paddlefish, Modrell, Lyne, et al., 2017; we also identified an otoferlin isoform maintained in ampullary organs but not neuromasts), Apba1 (Mint1), Rab3a and the pre‐synaptic organiser protein Nrxn3. Expression in both neuromasts and ampullary organs was also seen for a calcium‐independent synaptotagmin gene, *Syt14*, and *Dscaml1*, encoding a homophilic cell adhesion molecule that seems to be important for the final maturation of ribbon synapses in rod bipolar cells. Nrxn3 likely forms a synaptic complex with secreted Cbln18 at hair cell but not electroreceptor ribbon synapses, as *Cbln18* was expressed in neuromasts but not ampullary organs. Conversely, *Tulp1*, which is required for the development and function of ribbon synapses in photoreceptors, was expressed in ampullary organs but not neuromasts, suggesting Tulp1 may play similar roles in electroreceptors. Finally, *Pcp4* (encoding calmodulin regulator protein PCP4) was also expressed in sterlet neuromasts and ampullary organs. However, the relatively low expression level of *Pcp4* in ampullary organs versus neuromasts may suggest potential differences in PCP4 regulation of Ca^2+^‐calmodulin in electroreceptors versus hair cells. Overall, our gene expression analysis has given novel molecular insights into similarities and differences between ribbon synapses in electroreceptors and hair cells.

## AUTHOR CONTRIBUTIONS

A.S.C. led the project, performed most of the experiments, prepared some of the manuscript figures, and wrote the first draft of the manuscript. M.M. performed some of the experiments, prepared some of the manuscript figures and finalised the phylogenetic tree figures. D.B. undertook the phylogenetic analysis and provided the phylogenetic trees. T.A. performed some of the experiments and contributed to data interpretation. M.P. and D.G. were instrumental in enabling all work with sterlet embryos. C.V.H.B. conceived the project, provided guidance and helped to write the manuscript together with A.S.C. All authors read and commented on the manuscript.

## FUNDING INFORMATION

This work was supported by the Anatomical Society and by the Biotechnology and Biological Sciences Research Council (BBSRC: grant BB/P001947/1 to C.V.H.B.). A.S.C. was supported by a PhD research studentship from the Anatomical Society with additional funding from the Cambridge Philosophical Society. Additional support for M.M. was provided by the Isaac Newton Trust, University of Cambridge (grant 20.07[c] to C.V.H.B.) and by the School of the Biological Sciences, University of Cambridge. The work of M.P. was supported by the Ministry of Education, Youth and Sports of the Czech Republic projects CENAKVA (ID 90099) and Biodiversity (CZ.02.1.01/0.0/0.0/16_025/0007370) and by the Czech Science Foundation (22‐31141J).

## CONFLICT OF INTEREST STATEMENT

The authors have no conflicts of interest to declare.

## ETHICS STATEMENT

Sterlet animal work was reviewed and approved by the Animal Research Committee of the Research Institute of Fish Culture and Hydrobiology, Faculty of Fisheries and Protection of Waters, University of South Bohemia in České Budějovice, Vodňany, Czech Republic and the Ministry of Agriculture of the Czech Republic (MSMT‐12550/2016–3). Experimental fish were maintained according to the principles of the European Union (EU) Harmonized Animal Welfare Act of the Czech Republic, and Principles of Laboratory Animal Care and National Laws 246/1992 “Animal Welfare” on the protection of animals.

## RIGHTS RETENTION STATEMENT

This work was funded by a grant from the Biotechnology and Biological Sciences Research Council (BBSRC: BB/P001947/1). For the purpose of open access, the author has applied a Creative Commons Attribution (CC BY) licence to any Author Accepted Manuscript version arising.

## Supporting information


**Figure S1.** Phylogenetic analysis using MrBayes shows that the sterlet ortholog of the lateral line organ‐enriched paddlefish *cerebellin* transcript encodes Cbln18. Phylogenetic tree of cerebellin family amino acid sequences generated using MrBayes v.3.2.7 (Ronquist et al., 2012). Sequence names reflect the reference‐genome annotation and show the chromosomal (Chr) location of the gene if multiple cerebellin genes in that species share the same or similar annotation (e.g. sterlet [Ar] *Cbln1* on chromosomes 19, 20, 40 and 44, and *Cbln1‐like* on Chromosomes 39 and 54). Maximum support for the Cbln18 clade shows that the lateral line‐enriched paddlefish *cerebellin* transcript (Modrell, Lyne, et al., 2017) and its sterlet ortholog (highlighted in bold red font) encode Cbln18 and have been mis‐annotated as *Cbln3* in the respective reference genomes (paddlefish GCF_017654505.1; sterlet GCF_902713425.1). Further, the sterlet *Cbln20* ohnologs have been mis‐annotated in the reference genome as *Cbln1‐like* (chromosome 39) and *Cbln1* (chromosome 40). The tree also shows that Cbln3 (nested within the Cbln1 clade in this tree) is specific to mammals: all non‐mammalian sequences annotated as *Cbln3* cluster in other clades. GenBank accession numbers for the sequences used (104 from vertebrates plus the single amphioxus cerebellin sequence) are given in Table S2. Species abbreviations: Ar, *Acipenser ruthenus*; Am, *Alligator mississippiensis*; Bf, *Branchiostoma floridae*; Cl, *Canis lupus*; Cm, *Callorhinchus milii*; Dr, *Danio rerio*; Ec, *Erpetoichthys calabaricus*; Gg, *Gallus gallus*; Hs, *Homo sapiens*; Lo, *Lepisosteus oculatus*; Mm, *Mus musculus*; Ol, *Oryzias latipes*; Om, *Oncorhynchus mykiss*; Ps, *Polyodon spathula*; Pa, *Protopterus annectens*; Sc, *Scyliorhinus canicula*; Xt, *Xenopus tropicalis*.
**Figure S2.** Phylogenetic analysis using PhyloBayes shows that the sterlet ortholog of the lateral line organ‐enriched paddlefish *cerebellin* transcript encodes Cbln18. Phylogenetic tree of cerebellin family amino acid sequences generated using PhyloBayes MPI v1.8c (Lartillot et al., 2013). Sequence names reflect the reference‐genome annotation and show the chromosomal (Chr) location of the gene if multiple *cerebellin* genes in that species share the same or similar annotation (e.g. sterlet [Ar] *Cbln1* on chromosomes 19, 20, 40 and 44, and *Cbln1‐like* on chromosomes 39 and 54). Maximum support for the Cbln18 clade shows that the lateral line‐enriched paddlefish *cerebellin* transcript (Modrell, Lyne, et al., 2017) and its sterlet ortholog (highlighted in bold red font) encode Cbln18 and have been mis‐annotated as *Cbln3* in the respective reference genomes (paddlefish GCF_017654505.1; sterlet GCF_902713425.1). Further, the sterlet *Cbln20* ohnologs have been mis‐annotated in the reference genome as *Cbln1‐like* (chromosome 39) and *Cbln1* (chromosome 40). The tree also shows that Cbln3 is specific to mammals: all non‐mammalian sequences annotated as *Cbln3* cluster in other clades. GenBank accession numbers for the sequences used (104 from vertebrates plus the single amphioxus cerebellin sequence) are given in Table S2. Species abbreviations: Ar, *Acipenser ruthenus*; Am, *Alligator mississippiensis*; Bf, *Branchiostoma floridae*; Cl, *Canis lupus*; Cm, *Callorhinchus milii*; Dr, *Danio rerio*; Ec, *Erpetoichthys calabaricus*; Gg, *Gallus gallus*; Hs, *Homo sapiens*; Lo, *Lepisosteus oculatus*; Mm, *Mus musculus*; Ol, *Oryzias latipes*; Om, *Oncorhynchus mykiss*; Ps, *Polyodon spathula*; Pa, *Protopterus annectens*; Sc, *Scyliorhinus canicula*; Xt, *Xenopus tropicalis*.

Table S1.

Table S2.

## Data Availability

The publication and associated supplementary figures include representative example images of larvae from each experiment. Additional data underlying this publication consist of further images of these and other larvae from each experiment. Public sharing of these images is not cost‐efficient, but they are available from the corresponding author upon reasonable request. Previously published sterlet transcriptome assemblies (from pooled Stages 40–45 sterlet heads; Minařík et al., 2024) are available at DDBJ/EMBL/GenBank under the accessions GKLU00000000 (https://www.ncbi.nlm.nih.gov/nuccore/GKLU00000000) and GKEF01000000 (https://www.ncbi.nlm.nih.gov/nuccore/GKEF00000000.1). Previously published paddlefish RNA‐seq data (from pooled paddlefish opercula and fin tissue at Stage 46; Modrell, Lyne, et al., 2017) are available via the NCBI Gene Expression Omnibus (GEO) database (https://www.ncbi.nlm.nih.gov/geo/) under accession code GSE92470.
