# Supplementary material for: Molecular insights into electroreceptor ribbon synapses from differential gene expression in sturgeon lateral line organs
Source: J Anat. 2025 Nov 21;248(5):784–805. doi: 10.1111/joa.70061 (PMC13069148; doi:10.1111/joa.70061)
Supplement: Supplementary file 1 — Figure S1. Phylogenetic analysis using MrBayes shows that the sterlet ortholog of the lateral line organ‐enriched paddlefish cerebellin transcript encodes Cbln18. Phylogenetic tree of cerebellin family amino acid sequences generated using MrBayes v.3.2.7 (Ronquist et al., 2012). Sequence names reflect the reference‐genome annotation and show the chromosomal (Chr) location of the gene if multiple cerebellin genes in that species share the same or similar annotation (e.g. sterlet [Ar] Cbln1 on chromosomes 19, 20, 40 and 44, and Cbln1‐like on Chromosomes 39 and 54). Maximum support for the Cbln18 clade shows that the lateral line‐enriched paddlefish cerebellin transcript (Modrell, Lyne, et al., 2017) and its sterlet ortholog (highlighted in bold red font) encode Cbln18 and have been mis‐annotated as Cbln3 in the respective reference genomes (paddlefish GCF_017654505.1; sterlet GCF_902713425.1). Further, the sterlet Cbln20 ohnologs have been mis‐annotated in the reference genome as Cbln1‐like (chromosome 39) and Cbln1 (chromosome 40). The tree also shows that Cbln3 (nested within the Cbln1 clade in this tree) is specific to mammals: all non‐mammalian sequences annotated as Cbln3 cluster in other clades. GenBank accession numbers for the sequences used (104 from vertebrates plus the single amphioxus cerebellin sequence) are given in Table S2. Species abbreviations: Ar, Acipenser ruthenus; Am, Alligator mississippiensis; Bf, Branchiostoma floridae; Cl, Canis lupus; Cm, Callorhinchus milii; Dr, Danio rerio; Ec, Erpetoichthys calabaricus; Gg, Gallus gallus; Hs, Homo sapiens; Lo, Lepisosteus oculatus; Mm, Mus musculus; Ol, Oryzias latipes; Om, Oncorhynchus mykiss; Ps, Polyodon spathula; Pa, Protopterus annectens; Sc, Scyliorhinus canicula; Xt, Xenopus tropicalis. Figure S2. Phylogenetic analysis using PhyloBayes shows that the sterlet ortholog of the lateral line organ‐enriched paddlefish cerebellin transcript encodes Cbln18. Phylogenetic tree of cerebellin family amino acid [file JOA-248-784-s002.pdf]

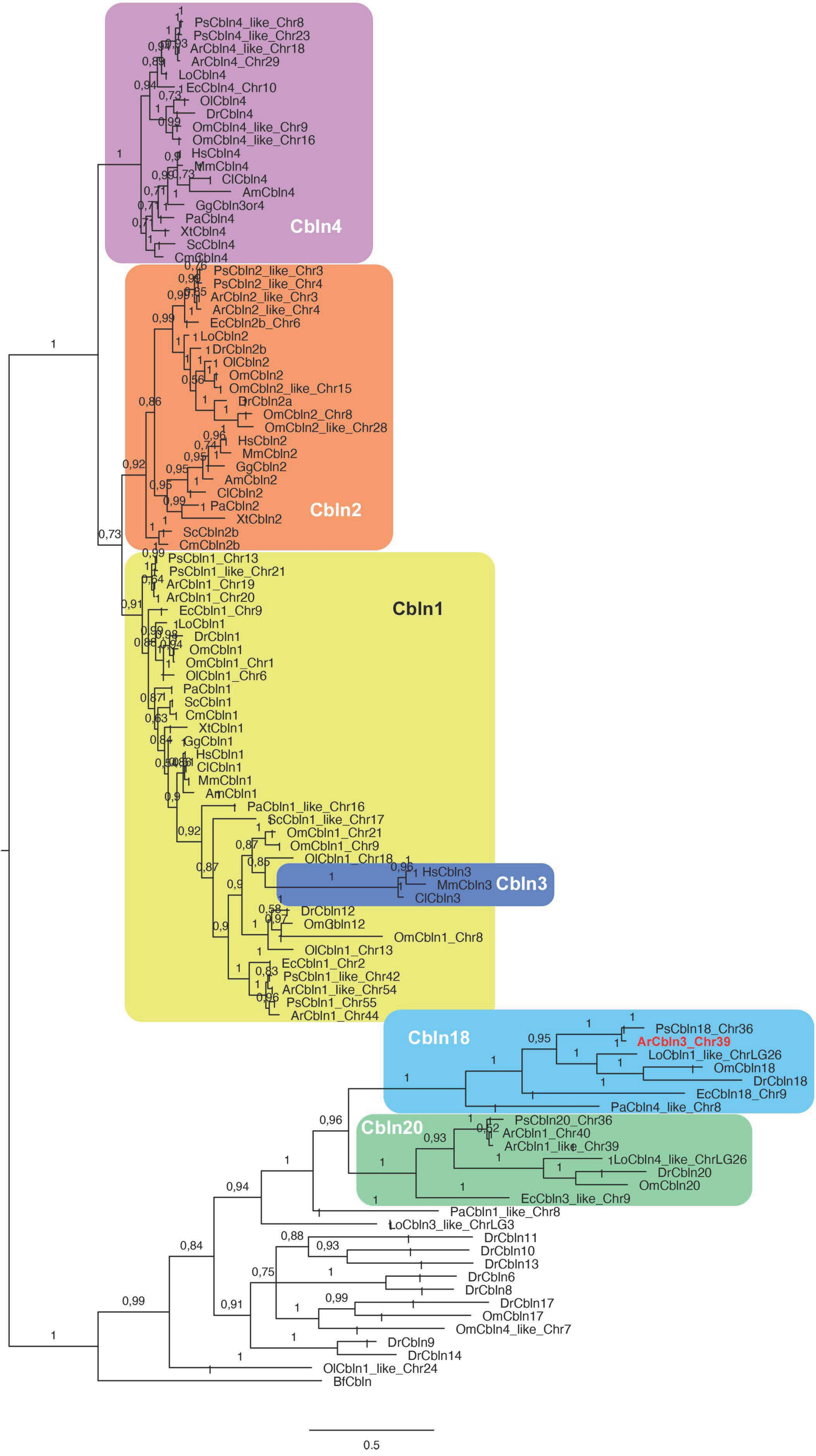

Supplementary Figure S1: Phylogenetic analysis using MrBayes shows that the sterlet ortholog of the lateral line organ-enriched paddlefish *cerebellin* transcript encodes Cbln18. Phylogenetic tree of cerebellin family amino acid sequences generated using MrBayes v.3.2.7 (Ronquist et al., 2012). Sequence names reflect the reference-genome annotation and show the chromosomal (Chr) location of the gene if multiple cerebellin genes in that species share the same or similar annotation (e.g., sterlet [Ar] *Cbln1* on chromosomes 19, 20, 40, and 44, and *Cbln1-like* on chromosomes 39 and 54). Maximum support for the Cbln18 clade shows that the lateral line-enriched paddlefish *cerebellin* transcript (Modrell et al., 2017) and its sterlet ortholog (highlighted in bold red font) encode Cbln18 and have been mis-annotated as *Cbln3* in the respective reference genomes (paddlefish GCF_017654505.1; sterlet GCF_902713425.1). Further, the sterlet *Cbln20* ohnologs have been mis-annotated in the reference genome as *Cbln1-like* (chromosome 39) and *Cbln1* (chromosome 40). The tree also shows that Cbln3 (nested within the Cbln1 clade in this tree) is specific to mammals: all non-mammalian sequences annotated as *Cbln3* cluster in other clades. GenBank accession numbers for the sequences used (104 from vertebrates plus the single amphioxus cerebellin sequence) are given in Supplementary Table S2. Species abbreviations: Ar, *Acipenser ruthenus;* Am, *Alligator mississippiensis;* Bf, *Branchiostoma floridae;* Cl, *Canis lupus;* Cm, *Callorhinchus milii;* Dr, *Danio rerio;* Ec, *Erpetoichthys calabaricus;* Gg, *Gallus gallus;* Hs, *Homo sapiens;* Lo, *Lepisosteus oculatus;* Mm, *Mus musculus;* Ol, *Oryzias latipes;* Om, *Oncorhynchus mykiss;* Ps, *Polyodon spathula;* Pa, *Protopterus annectens;* Sc, *Scyliorhinus canicula;* Xt, *Xenopus tropicalis.*

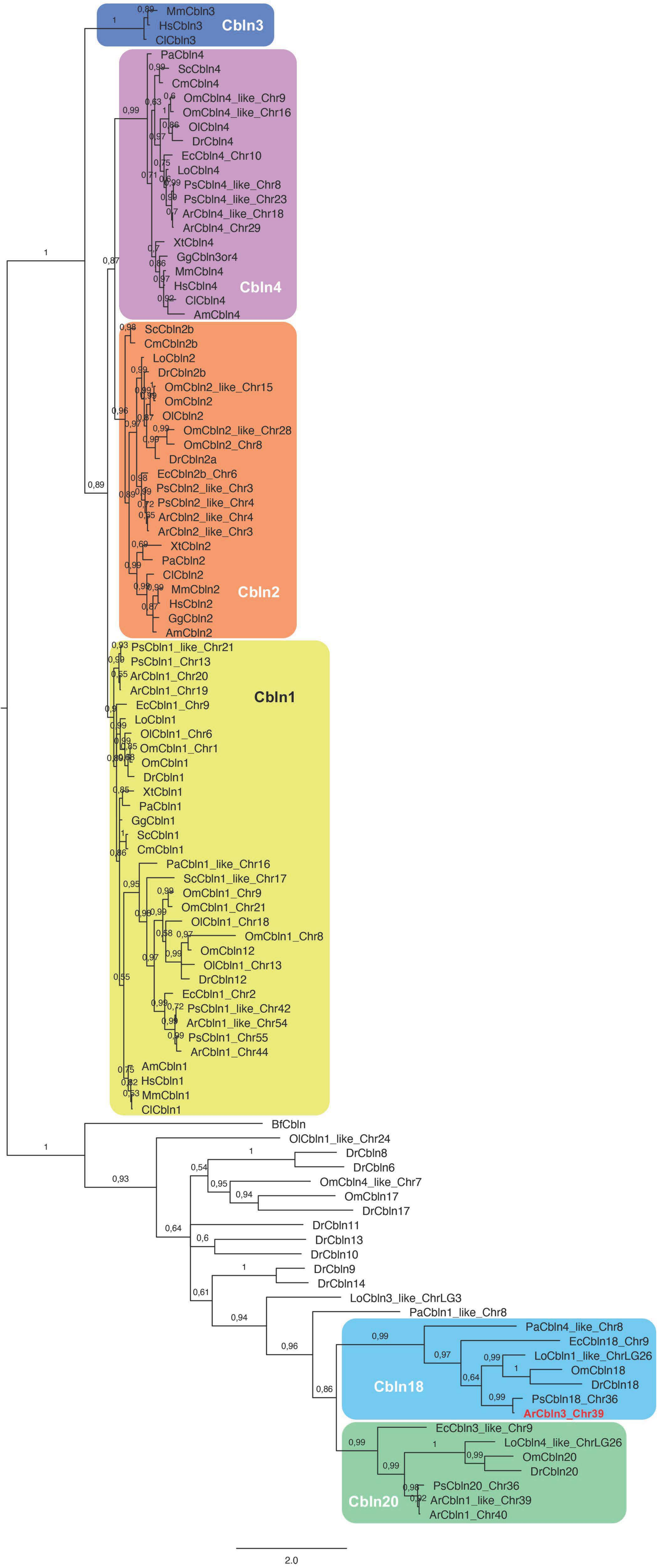

Supplementary Figure S2: Phylogenetic analysis using PhyloBayes shows that the sterlet ortholog of the lateral line organ-enriched paddlefish *cerebellin* transcript encodes Cbln18. Phylogenetic tree of cerebellin family amino acid sequences generated using PhyloBayes MPI v1.8c (Lartillot et al., 2013). Sequence names reflect the reference-genome annotation and show the chromosomal (Chr) location of the gene if multiple *cerebellin* genes in that species share the same or similar annotation (e.g., sterlet [Ar] *Cbln1* on chromosomes 19, 20, 40, and 44, and *Cbln1-like* on chromosomes 39 and 54). Maximum support for the Cbln18 clade shows that the lateral line-enriched paddlefish *cerebellin* transcript (Modrell et al., 2017) and its sterlet ortholog (highlighted in bold red font) encode Cbln18 and have been mis-annotated as *Cbln3* in the respective reference genomes (paddlefish GCF_017654505.1; sterlet GCF_902713425.1). Further, the sterlet *Cbln20* ohnologs have been mis-annotated in the reference genome as *Cbln1-like* (chromosome 39) and *Cbln1* (chromosome 40). The tree also shows that Cbln3 is specific to mammals: all non-mammalian sequences annotated as *Cbln3* cluster in other clades. GenBank accession numbers for the sequences used (104 from vertebrates plus the single amphioxus cerebellin sequence) are given in Supplementary Table S2. Species abbreviations: Ar, *Acipenser ruthenus;* Am, *Alligator mississippiensis;* Bf, *Branchiostoma floridae;* Cl, *Canis lupus;* Cm, *Callorhinchus milii;* Dr, *Danio rerio;* Ec, *Erpetoichthys calabaricus;* Gg, *Gallus gallus;* Hs, *Homo sapiens;* Lo, *Lepisosteus oculatus;* Mm, *Mus musculus;* Ol, *Oryzias latipes;* Om, *Oncorhynchus mykiss;* Ps, *Polyodon spathula;* Pa, *Protopterus annectens;* Sc, *Scyliorhinus canicula;* Xt, *Xenopus tropicalis*.
